# Fitness Costs of Two Maize Lepidopteran Pests Fed on *Bacillus thuringiensis* (Bt) Diets Enriched with Vitamins A and C

**DOI:** 10.3390/insects12080718

**Published:** 2021-08-11

**Authors:** Carmen López, Pilar Muñoz, Daniela Zanga, Patricia Sarai Girón-Calva, Matilde Eizaguirre

**Affiliations:** 1 Department of Crop and Forest Sciences, University of Lleida-Agrotenio Center, Av. Al. Rovira Roure 191, 25198 Lleida, Spain; carmen.lopez@udl.cat (C.L.); pilarm@pvcf.udl.cat (P.M.); sarai.giron@pvcf.udl.cat (P.S.G.-C.); 2 Laboratori de Sanitat Vegetal, Departament d’Agricultura, Ramadera i Pesca, Generalitat de Catalunya, 25198 Lleida, Spain; daniela.zanga12345@gmail.com

**Keywords:** biofortified Bt crops, vitamin A, β-carotene, vitamin C, ascorbic acid, AsA, phytophagous, maize, antioxidants enzymes, *Helicoverpa armigera*, *Mythimna unipuncta*, Lepidoptera, Noctuidae

## Abstract

**Simple Summary:**

Biotechnologists are designing new transgenic plants enriched with micronutrients and vitamins that are resistant to insects. These new plants could favor the development of some pest insects. This work aims to discover the effect of adding two vitamins, A and C, to insect diets prepared with Bt and no-Bt maize in two maize insect pests. *M. unipuncta* was less sensitive to the toxin, although ingestion of the Bt diet resulted in longer larval development and lower pupal weight, which were not mitigated by any of the vitamins. However, the two vitamins reduced the mortality of *H. armigera* larvae fed on the Bt diet. In addition, we found evidence of the antioxidant function of vitamin A. The results obtained here indicate that crops enriched with these vitamins will hardly favor the development of *H. armigera* and suggest that they do not affect *M. unipuncta’*s development at all.

**Abstract:**

Serious malnutrition problems occur in developing countries where people’s diets are mainly based on staple crops. To alleviate this, high-production crops are being developed that are better adapted to climate change, enriched in micronutrients and vitamins, or resistant to pests. In some cases, new varieties have been developed with several of the characteristics mentioned above, such as biofortified and pest-resistant crops. The development of biofortified *Bacillus thuringiensis* (Bt) crops raises the question of whether vitamin enrichment of Bt crops can in any way favor those pests that are not very susceptible to the Bt toxin that feed on these crops, such as *Helicoverpa armigera* (Hübner) or *Mythimna unipuncta* (Haworth) (Lepidoptera: Noctuidae). In this study, the response to a Bt diet enriched with vitamins A (β-carotene) and C (ascorbic acid) was somewhat different between the two species. *M. unipuncta* was less sensitive to the toxin than *H. armigera*, although the ingestion of the Bt diet resulted in oxidative stress (longer larval development and lower pupal weight) which was not mitigated by the vitamins. However, the two vitamins reduced the mortality of *H. armigera* larvae fed on a Bt-enriched diet; in addition, ß-carotene reduced the activity of the antioxidant glutathione S-transferase (GST) of both species, suggesting it has an antioxidant role. The results obtained here indicate that biofortified Bt crops will not favor the development of *H. armigera* very much and will not affect *M. unipuncta’*s development at all, although the effect of the increase in vitamins may be very variable and should be studied for each specific phytophagous.

## 1. Introduction

Malnutrition is a serious problem in many developing countries where people’s diets are mainly based on staple crops (rice, sorghum, or maize) that are poor in micronutrients (vitamins, iron, and zinc) [1]. The 2017 Global Report on Food Crises [2] revealed that hunger and malnutrition are increasing, with approximately 108 million individuals in 48 countries at risk or in severe food insecurity. For these reasons, research on new staple crops with higher yields, better adaptation to climate change, and improved ability to produce vitamins and micronutrients has been considered a challenge to mitigate world hunger. Genetically modified crops with these traits have been considered suitable to address these challenges [3].

Transgenic crops incorporating genes from the entomopathogenic bacterium *Bacillus thuringiensis* Berliner (e.g., Bt maize with Cry1Ab toxin) have been grown commercially since 1996. In 2018, twenty-six countries (21 developing and 5 industrialised countries) planted 191.7 million hectares of transgenic crops, most of which had two or more Bt toxins or were improved with herbicide tolerance [4]. Many new, biofortified, conventional or transgenic staple crops with enhanced nutritional traits, such as the accumulation of high levels of β-carotene and vitamin C (AsA), are under development or nearing commercialisation [5,6]. Some examples of these developing biofortified transgenic crops are transgenic biofortified sorghum for nutritional improvement [7] or the ‘Golden’ rice, recently authorised for consumption in the Philippines [8]. The FAO supports a science-based evaluation system that objectively determines the benefits and risks of each new genetically modified organism (GMO). This calls for a cautious case-by-case approach to address legitimate concerns for the biosafety of each product or process before its release [9].

The extended growth in a region of biofortified staple crops with increased production of several vitamins raises the so far little-studied question of how the phytophagous insects that feed on these enriched crops will respond in terms of development and mortality. In short, will these crops increase the populations of phytophagous pests?

Vitamins A and C have important roles in insect structure, behaviour, and physiology [10]. Among other functions, they serve as antioxidants and can contribute to the immune response of arthropods by scavenging reactive oxygen species [10]. Studies with insects have shown that dietary carotenoids can either decrease [11,12,13] or increase [14] the toxicity of some compounds. Insects also have enzymatic antioxidant systems that are necessary because the diet of phytophagous insects contains a large number of pro-oxidant molecules [10]. Antioxidant enzymes, such as catalase (CAT), glutathione S-transferase (GST), and superoxide dismutase (SOD) may play a very important role in the detoxification of reactive oxygen species (ROS) produced by xenobiotics and many plant-derived allelochemicals. Few studies have linked Bt infection with increased ROS levels in larvae. However, Dubovskiy [15] demonstrated that infection of *Galleria* *mellonella* larvae with Bt increased oxidative stress as well as the levels of antioxidant enzymes SOD and GST in the larval midgut as a consequence of the infection.

Therefore, insects that feed on transgenic Bt plants biofortified with vitamins can respond to the oxidative stress triggered by the bacterium in two ways: they can either increase the production of antioxidant enzymes or take advantage of the antioxidant effect of the vitamins. Few studies have been devoted to the analysis of the interactions of these two approaches (which might have a synergistic or antagonistic interaction inside larval body) with the Cry1A toxin.

Little is known about the effects of biofortified Bt crops on phytophagous insects, and how carotenoid or vitamin enhancement in host plants may affect their survival, development, and/or behaviour. Zanga et al. [13] found that in larvae of the Bt target pest *Ostrinia nubilalis* (Hübner) (Lepidoptera: Crambidae) the addition of β-carotene to a Bt diet moderated the effectiveness of Bt toxin, reducing larval mortality. However, Girón-Calva et al. [14] found that neonate larvae of *O. nubilalis* fed on Bt diets supplemented with β-carotene showed higher mortality. Zanga et al. [13] related this effect to the activity of three enzymes implicated in detoxification mechanisms, CAT, SOD, and GST. Similar studies could be particularly interesting for non-target caterpillars that are poorly susceptible to the Bt toxin, but could be favoured by the increased content of vitamins in Bt maize plants.

In Europe, Bt maize with truncated genes of the entomopathogenic bacterium, *B. thuringiensis*, is mostly grown in Spanish and Portuguese areas where maize Lepidopteran borers are serious pests. Bt is selectively toxic to some insects, many of which belong to Lepidoptera, in which the degree of susceptibility to the toxin differs according to the species [16,17]. The only Bt maize cultivated in the EU, which contains the transformation event MON810 (Cry1Ab; Monsanto Company, St. Louis, MO, USA), efficiently controls the two main maize borers present in the EU, namely the European corn borer (*O. nubilalis*), and the Mediterranean Corn Borer (*Sesamia nonagrioides* Lefèbvre (Lepidoptera: Noctuidae)) [18]. However, it has a much lower efficacy in controlling other secondary Lepidopteran maize pests, such as the true armyworm, *Mythimna unipuncta* Haworth, and the corn earworm, *Helicoverpa armigera* Hübner (Lepidoptera: Noctuidae). The cultivation of this Bt crop could result is an increased occurrence of the secondary pests which are less susceptible to Bt.

*M. unipuncta* is a polyphagous insect and an important pest of graminaceous crops in Europe [19] and North America [20]. *H. armigera* is a serious cosmopolitan insect pest whose larvae are polyphagous [21], and it has a high ability to develop resistance to insecticides [22] and to the Bt toxin [23]. Eizaguirre et al. [24] observed that the larvae of both species could survive and complete their development even when feeding on Bt maize and, therefore, ingesting Bt toxins. The introduction of biofortified Bt crops could improve the fitness of these two low-susceptibility species when feeding on these crops.

The objectives of the current study were to determine whether vitamins A (β–carotene) and C (ascorbic acid: AsA) added to a Bt diet improved the survival and development of each of the species with a low susceptibility to the Bt toxin, *H. armigera* and *M. unipuncta*. A second objective was to determine whether three different groups of antioxidant enzymes related to oxidative stress (CAT, SOD, and GST) might be involved in the low susceptibility to the toxin by comparing the responses of both species to the ingestion of the Bt toxin with or without β-carotene and AsA.

## 2. Materials and Methods

### 2.1. Insects

Two Lepidopteran species were used in this study, *H. armigera* and *M. unipuncta*. Populations of both insects were established in the lab from individuals collected in the fields (Lleida, Spain (GPS coordinates 41°37′39.1″ N, 0°35′39.5″ E). Larvae of *H. armigera* were collected in alfalfa, from summer to autumn in non-Bt maize fields. Adults of *M. unipuncta* were collected in September in light traps close to non-Bt maize fields.

The populations of both species were maintained at 25 °C under high humidity (>60%) and a 16:8 h light: dark (LD) photoperiod, and were renewed for each experiment, so that each new experiment was carried out with the laboratory’s F1. Laboratory larvae were reared on a semi-artificial diet [25]. Once they reached the adult stage, they were nourished with a sugar solution (10%). Females and males were placed in cages for mating and oviposition. Three cages with 6 females and 5 males were established for *H. armigera* and two cages with the same number (8–10) of females and males for *M. unipuncta*. The oviposition substrate for *H. armigera* was cotton while for *M. unipuncta* it consisted of corn plants. Laboratory-born larvae were used in this study. Since wild populations were collected at different periods, experiments with *H. armigera* an *M. unipuncta* were carried out at substantially different times.

### 2.2. Diets

Semi-artificial diets used in the experiments for insect rearing were modified in composition from the diet of [25] to include Cry1Ab insecticidal protein, different amounts of ascorbic acid (AsA), and of β-carotene (denoted as β). The basal diet contained lyophilized maize leaves (11.1% weight: w), brewer’s yeast (3.0% w), wheat germ (3.0% w), ascorbic acid (AsA, 0.5% w), sorbic acid (0.2% w), agar-agar (1.6% w), and water (80.6% w). As the common diet used to rear Lepidoptera in our laboratory contains 0.5% in weight of AsA [25], the diets used in the experiments were without, with 0.5% or with double amount (1%) of AsA for the tests. B-carotene amounts were chosen from the work of Zanga et al. [13]. Table 1 shows the qualitative and quantitative diet variants used for the experimental design of this study. For each treatment, the diet was prepared twice or, in some cases, thrice, since the available amount of diet was not enough to complete the experiment.

### 2.3. Plant Material

Cry1Ab insecticidal protein was incorporated into artificial diets by adding freeze-dried material from the leaves of commercial Bt maize DKC6667Y (Cry1Ab) MON810 while non-Bt maize DKC6666 (isogenic) was added to the non-Bt diets. Leaves from Bt and non-Bt maize had been collected in the summer of 2017 in field areas near Lleida in unsprayed plants. Leaves were cut into small strips and the main veins were removed. The material was later freeze-dried in a vacuum drier (Gamma 2–16 LSC plus, CHRIST, Osterode am Harz, Germany) and ground in a Thermomix^®^ until a fine powder was obtained. The lyophilized material was kept at −80 °C until use. The content of Cry1A insecticidal protein in Bt and Bt-β diets was verified using the Agdia Bt-Cry1Ab/Cry1Ac kit (Agdia Inc., Elkhart, IN, USA). For calibration, Cry1Ab standards at 100, 75, 50, 25, 15, 8, and 2 ng/mL were used. Measurements were made with a VICTOR3 Multilabel Plate Counter (PerkinElmer Life and Analytical Science, Madrid, Spain) at 650 nm.

ELISA analyses indicated that the toxin content in Bt diets amounted to 9 µg of Cry1Ab protein per gram of diet. This amount is similar to the average toxin content in a maize plant developed in the field [26].

### 2.4. Development Studies

Newly hatched larvae were provided with a semi-artificial diet [25] until their moult to the sixth larval instar (L_6_). Newly moulted larvae of the sixth instar developed under long day (16:8, LD) photoperiodic conditions were fed with the different experimental diets (Table 1). Larvae from each cage (different layings) were randomly divided between the twelve experimental conditions until reaching the necessary sample size in each condition. The effects of Bt, β-Carotene, and AsA on larval development, pupal weight, and mortality of two species, *H. armigera* and *M. unipuncta*, were studied. At least 20 (between 20 and 35) larvae were used per diet. Larvae were checked daily until pupation or death. When larvae pupated, the weight of pupae and the L_6_ developmental duration (in days) were recorded.

### 2.5. Enzymatic Studies

The activity of superoxide dismutase (SOD), catalase (CAT), and glutathione S-transferase (GST) enzymes involved in the detoxification processes in the insect midgut was measured. Between 8 and 24 newly moulted caterpillars of 6th instars (L_6_0) were used for each treatment. Each larva was fed with a cube c.a. 1cm^3^ of one experimental semi-artificial diet (Table 1) for 1 d and then immediately frozen in liquid nitrogen to be used for enzyme activity analysis (L_6_1).

To determine SOD and CAT activity, entire larval body samples were homogenized by sonication in PBS-Tween (0.5 mL PBS-Tween per 100 mg of tissue), followed by centrifugation at 10,000× *g* for 15 min at 4 °C. Supernatants were used for determinations according to the manufacturer’s instructions. The sensitivity of the assay for SOD was 0.044 U/mL (K028-H1; Arbor Assays, Ann Arbor, MI, USA); for CAT, it was 0.052 U/mL (K033-H1; Arbor Assays). The unit (U/mL) indicates the units of SOD/catalase activity per milliliter, normalized for protein concentration. Protein levels were measured using the Bradford method [27], based on the principle of protein–dye binding.

For GST activity, entire larval body samples were homogenized by sonication in 110 µL of HEPES buffer 0.05 M (pH 7), followed by centrifugation at 10,000× *g* for 15 min at 4 °C. Supernatants were then collected, and 30 µL of each sample was pipetted in duplicate onto a black enzyme-linked immune sorbent assay (ELISA) plate. To each well, 170 µL of the following mix was added: 3 mM GSH (cofactor), 0.3 mM monochlorobimane, and 50 mM HEPES buffer, pH 7. The plate was incubated at room temperature for 20 min in the dark. Fluorescence was measured at 380 nm excitation and 465 nm emission with an Infinite M nano + Plate Counter (TECAN Group Ltd., Männenord, Switzerland). Values are expressed as units of fluorescence per mg of protein per minute.

### 2.6. Statistical Analysis

Neither diet nor the cage used to supply insects had a significant (*p* < 0.05) influence on the measured variables and therefore these factors were no longer considered in the analysis.

To test the effect of each factor (Bt, β-carotene, and AsA) on development duration of the last instar, on pupal weight and on the activity of larval enzymes (SOD, CAT GST) a three-way ANOVA was used. *p* < 0.05 was considered significant. When needed, log transformation of data was applied to variables based on the Boxcox’s lambda magnitude. A Tuckey’s test was used for least square means comparison. The mortality was calculated as percentage for each trial but analyzed using a generalized linear model with a binomial distribution.

All Statistical tests were carried out using JMP^®^ Pro 15.2.0 statistical software version [28].

The statistical analysis done and the data used for them can be found in the Supplementary Materials.

## 3. Results

### 3.1. Effect of Bt Toxin, β-Carotene and Ascorbic Acid (Vitamins A and C) on Larval Development Duration and Pupal Weight

**Figure 1:**
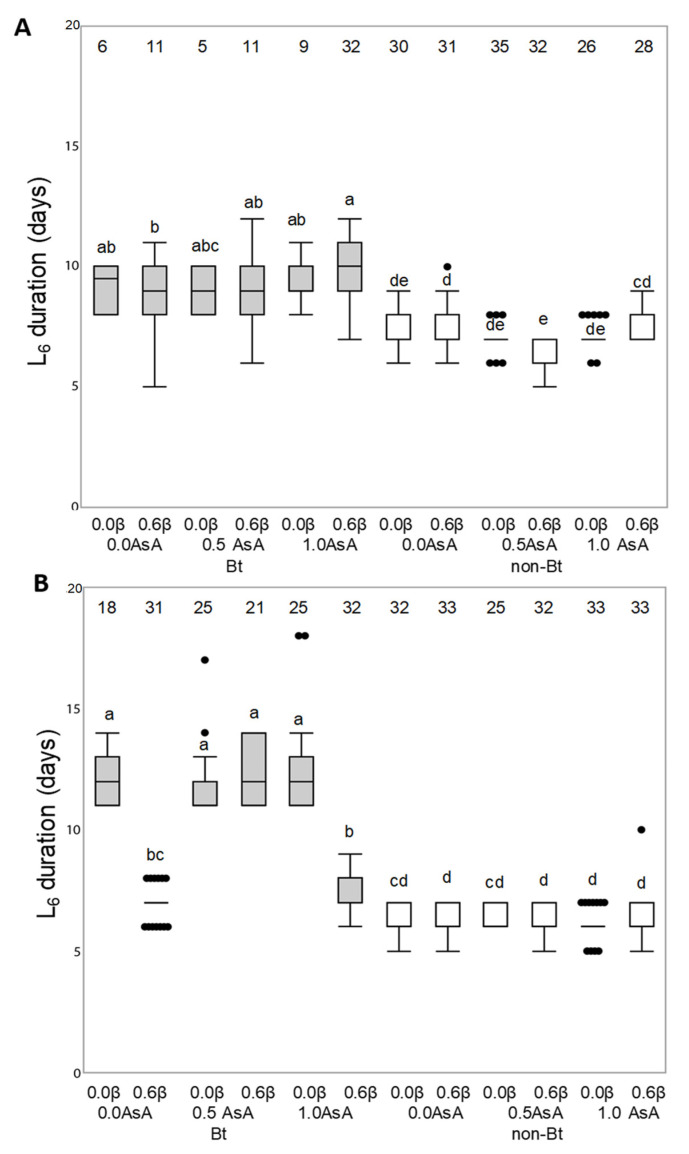
Effect of the addition of vitamins A (β-carotene: 0 or 0.6% in weight) and C (ascorbic acid, AsA: 0, 0.5 or 1.0% in weight) to non-Bt or Bt diets on the development duration (in days) of the last instar of *Helicoverpa armigera* (**A**) and *Mythimna unipuncta* (**B**). non-Bt (white box) indicates diets prepared with lyophilized leaves of non-Bt maize while Bt (grey box) indicates diets prepared with lyophilized leaves of Bt maize. Numbers on the top of each boxplot indicate the number of insects used for statistical analysis (survivors). Statistical test: Three-way ANOVA (*p* < 0.005), means were compared by Tukey’s test. Different lowercase letters over the boxplots indicate differences in larval duration.

Figure 1 compares the development duration of the last instar of *H. armigera* and *M. unipuncta* fed on the non-Bt or Bt diets both with or without vitamin enrichment. The addition of vitamins had different effects in the two insects. The duration of the L6 instar of *H. armigera* larvae fed on the Bt diet resulted to be longer than that of the larvae fed on the non-Bt diet (F_254, 2_ = 2124.9344, *p* = 0.001) and the AsA vitamin also affected the duration depending on the dose (F_254, 2_ = 6.4173, *p* = 0.0019) (Figure 1A). The length of the L6 instar of *M. unipuncta* larvae was influenced by the interaction between the type of diet (Bt or non-Bt) and the presence of the two vitamins (Asa and β-carotene) (Bt *AsA *β F_328, 2_ = 95.01; *p* < 0.001). Vitamin addition did not affect the duration of 6th instar larvae fed on non-Bt diet, while for larvae fed on the Bt diet the lenght of the instar depended on the AsA*β interaction (F_328, 2_ = 63.3893; *p* < 0.001) (Figure 1B).

**Figure 2:**
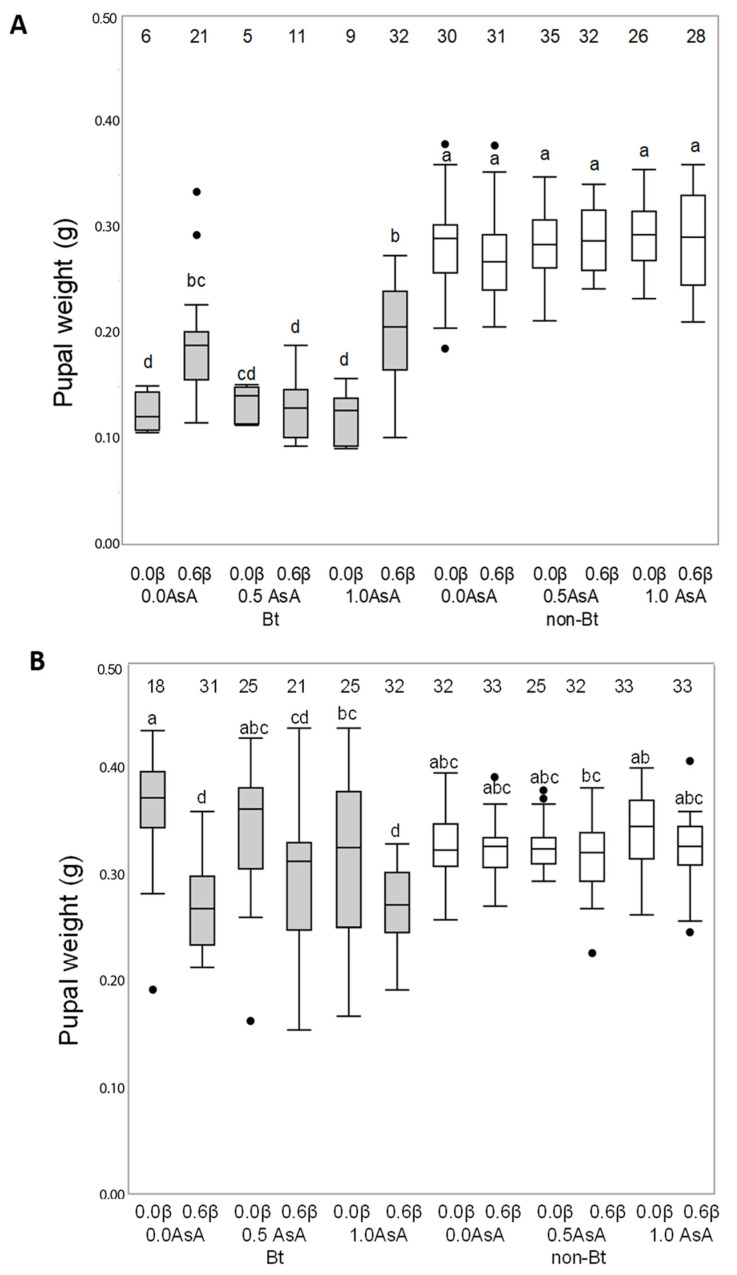
Effect of the addition of vitamins A (β-carotene: 0 or 0.6% in weight) and C (ascorbic acid, AsA: 0, 0.5 or 1.0% in weight) to non-Bt or Bt diets on the weight of the resulting pupae of *Helicoverpa armigera* (**A**) and *Mythimna unipuncta* (**B**). non-Bt (white box) indicates diets prepared with lyophilized leaves of non-Bt maize while Bt (grey box) indicates diets prepared with lyophilized leaves of Bt maize. Numbers on the top of each boxplot indicate the number of insects used for statistical analysis (survivors). Statistical test: Three-way ANOVA (*p* < 0.005), means were compared by Tukey’s test. Different lowercase letters over the boxplots indicate differences in pupal weight.

The weight of the resulting pupae of *H. armigera* and *M. unipuncta* larvae fed on the non-Bt or Bt diets is shown in Figure 2. A triple interaction between the type of diet (Bt or non-Bt) and the presence of the two vitamins (AsA and β-carotene) was observed for pupal weight in *H. armi*gera (F_253, 2_ = 6.1050; *p* = 0.0026). Vitamin addition did not affect the weight the of pupae of the larvae fed on non-Bt diet, but larvae fed on the Bt diet had lower pupal weight compared to larvae fed on the non-Bt diet and depended on the interaction between the vitamins (AsA and β-carotene) (F_253, 2_ = 3.0392; *p* < 0.0496) (Figure 2A). Pupal weight for *M. unipuncta* was also affected by the triple interaction between the type of diet (Bt or non-Bt) and the presence of the two vitamins (Asa and β-carotene) (F_328, 2_ = 3.7550; *p* = 0.024); the presence of β-carotene reduced pupal weight of larvae fed on the Bt diet (F_328, 2_ = 45.9346; *p* = 0.001) (Figure 2B).

### 3.2. Larval Mortality

Table 2 shows the percentage of mortality for each larval species when fed on the experimental diets. A small number of *M. unipuncta* larvae fed on Bt diet (with or without ascorbic acid and β-carotene) died and there were no significant differences in the % of mortality for larvae fed on non-Bt or Bt diets with the different amounts of vitamins (χ^2^_339,11_ = 19.829; *p* = 0.0477). However, mortality of *H. armigera* larvae fed on the Bt (44%) diet enriched with AsA or β-carotene was higher compared to that of larvae fed on the non-Bt one (6%). (χ^2^_371,11_ = 109.46; *p* < 0.0001).

**Figure 3:**
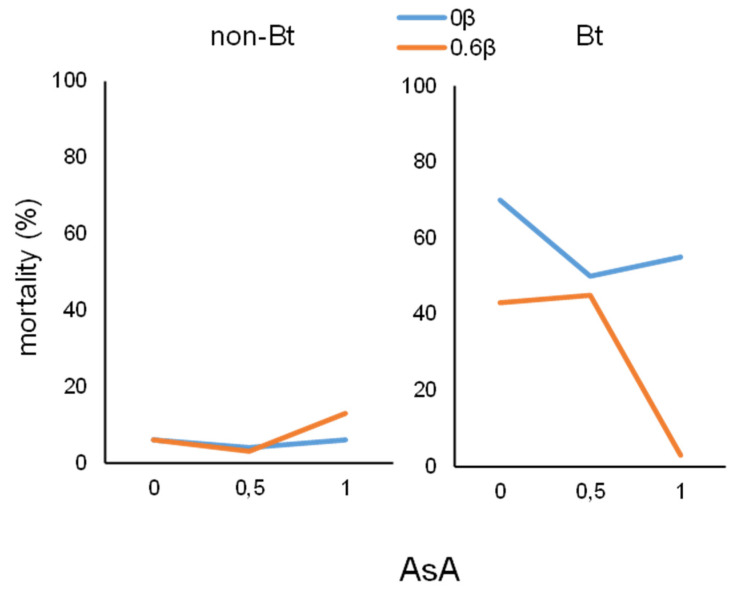
Mortality of *H. armigera* larvae fed on Bt and non-Bt diets with different amounts of vitamin C (ascorbic acid, AsA) and vitamin A (β-carotene).

Significant effects resulted to be: the triple interaction (χ^2^_371,2_ = 6.63; *p* = 0.0363; df = 2), the doubles ones Bt * AsA (χ^2^_371,2_ = 8.79; *p* = 0.012) and Bt*β (χ^2^_371,1_ = 1.87; *p* = 0.0139) and Bt (χ^2^_371,1_ = 22.13; *p* < 0.0001) (Figure 3). *H. armigera* larvae fed on non-Bt diets showed very low mortality (~10%) regardless of the vitamin content of the diets. When larvae were fed on Bt diets, mortality was higher if the diet did not contain β-carotene, while in the presence of β-carotene there was a reduction in mortality, especially in combination with the highest amount of AsA.

### 3.3. Antioxidant Enzymatic Activity

**Figure 4:**
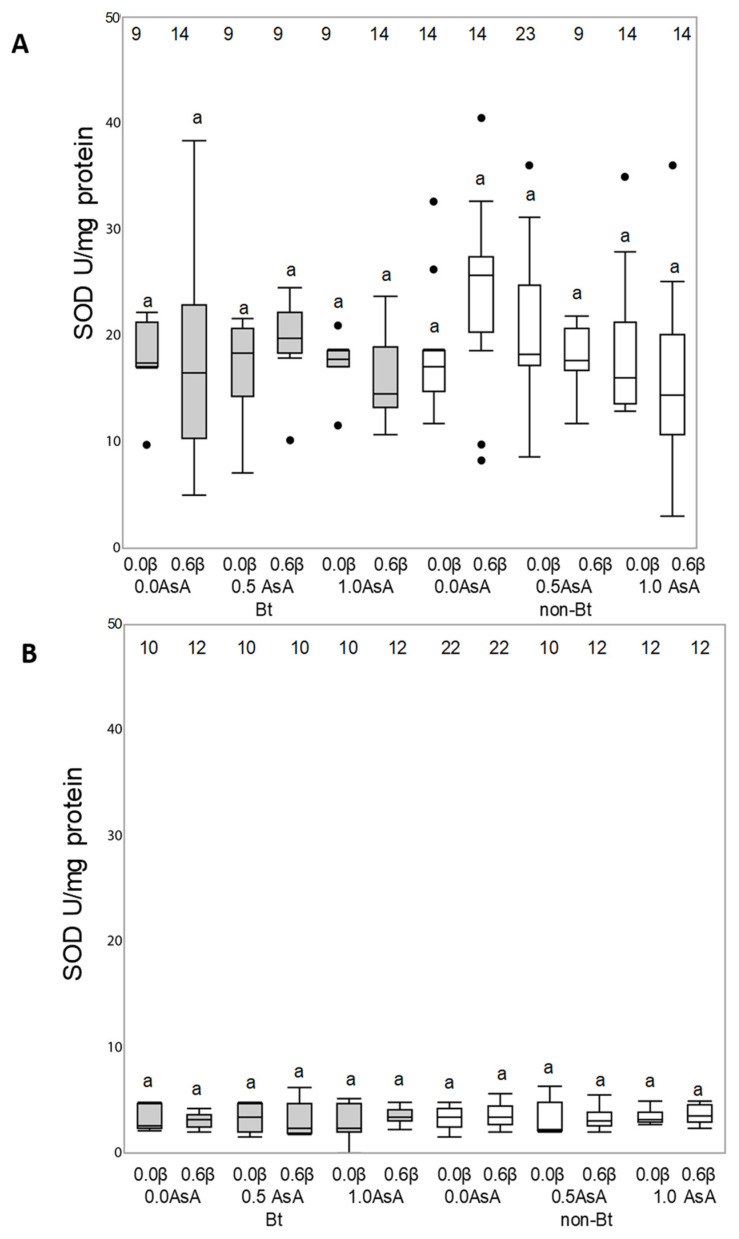
Responses of the antioxidant superoxide dismutase (SOD) enzyme of *Helicoverpa armigera* (**A**) and *Mythimna unipuncta* larvae (**B**) to the feeding on a non-Bt (white box) or Bt (grey box) diet enriched with vitamins A (β-carotene: 0 or 0.6% in weight) or C (ascorbic acid, AsA: 0, 0.5 or 1.0% in weight). Numbers on the top of each boxplot indicate the number of insects used for statistical analysis (survivors). Statistical test: Three-way ANOVA (*p* < 0.005) means were compared by Tukey’s test. Different letters over the boxplots indicate differences between each enzymatic response.

**Figure 5:**
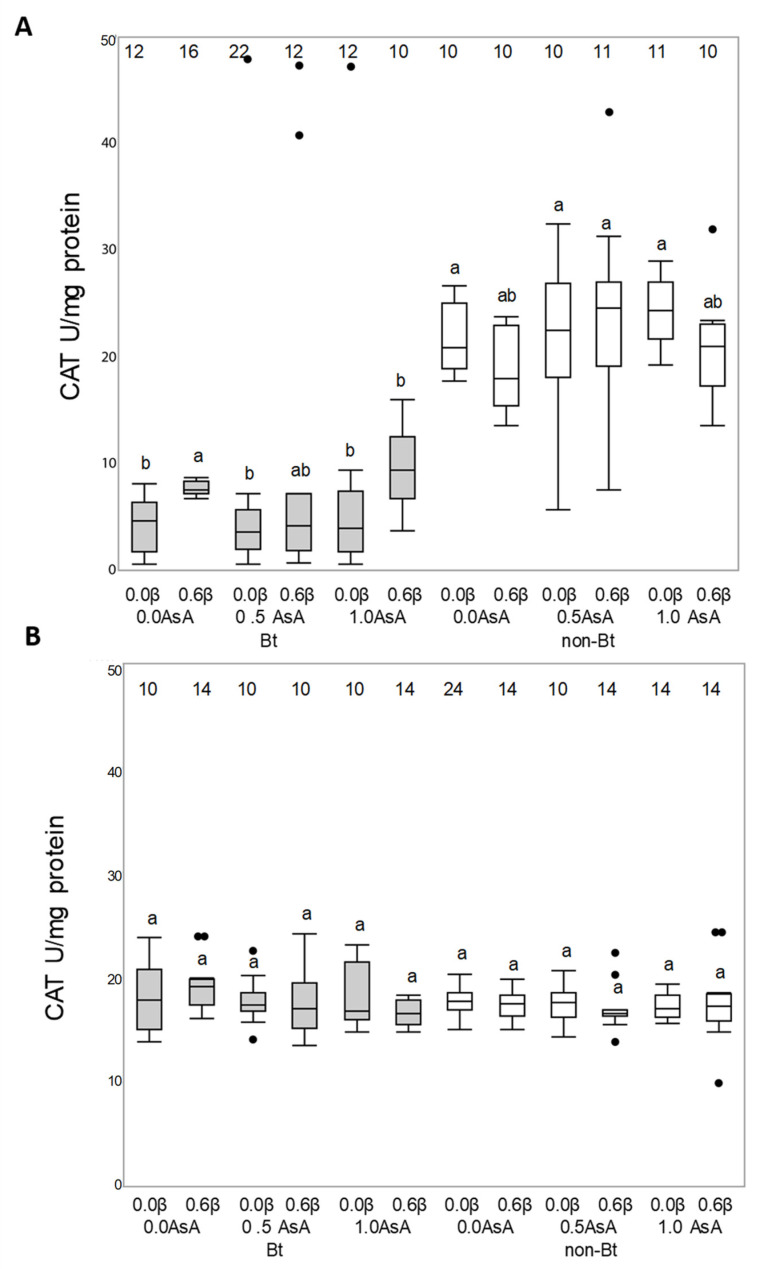
Responses of catalase (CAT) enzyme of *Helicoverpa armigera* (**A**) and *Mythimna unipuncta* larvae (**B**) to the feeding on a non-Bt (white box) or Bt (grey box) diet enriched with vitamins A (β-carotene: 0 or 0.6% in weight) or C (ascorbic acid, AsA: 0, 0.5 or 1.0% in weight). Numbers on the top of each boxplot indicate the number of insects used for statistical analysis (survivors). Statistical test: Three-way ANOVA (*p* < 0.005), means were compared by Tukey’s test. Different letters over the boxplots indicate differences between each enzymatic response.

**Figure 6:**
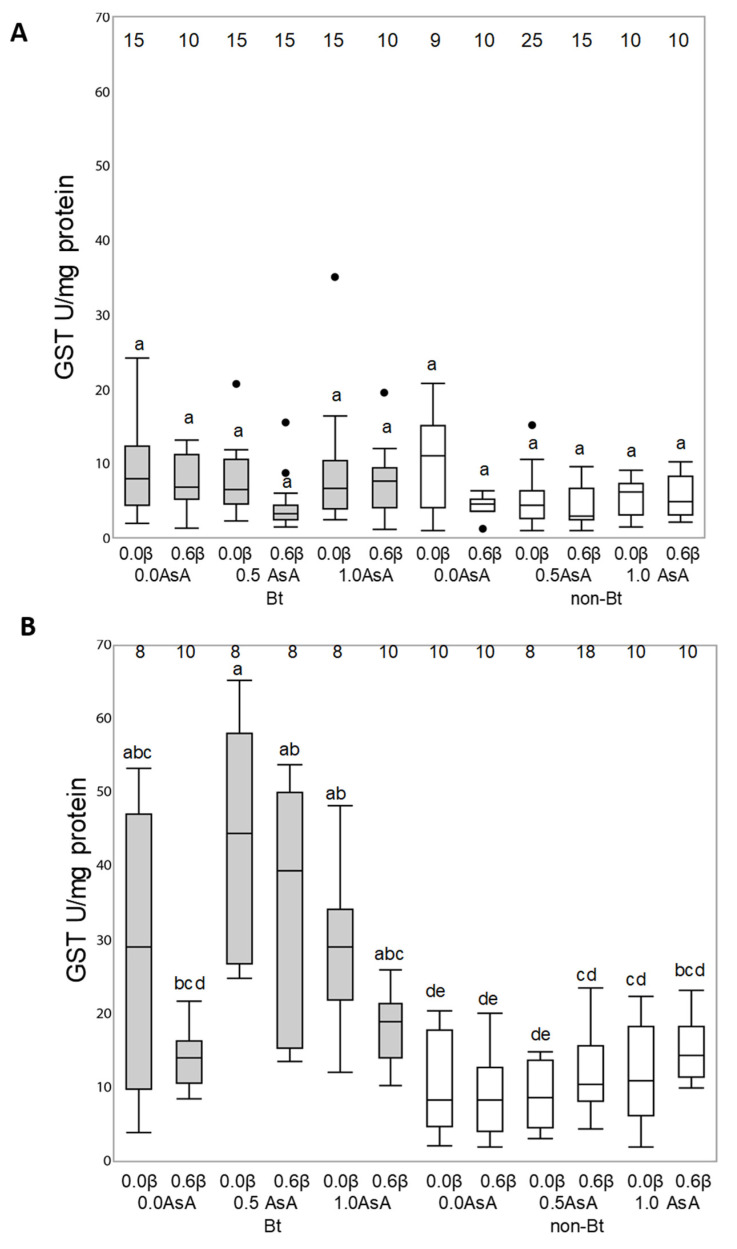
Responses of glutathione S-transferases (GST) enzyme in *Helicoverpa armigera* (**A**) and *Mythimna unipuncta* larvae (**B**) to the feeding on a non-Bt (white box) or Bt (grey box) diet enriched with vitamins A (β-carotene: 0 or 0.6% in weight) or C (ascorbic acid, AsA: 0, 0.5 or 1.0% in weight). Numbers on the top of each boxplot indicate the number of insects used for statistical analysis (survivors). Statistical test: Three-way ANOVA (*p* < 0.005), means were compared by Tukey’s test. Different letters over the boxplots indicate differences between each enzymatic response.

Antioxidant enzymatic activity of superoxide dismutase (SOD), catalase (CAT) and glutathione S-transferase (GST) enzymes of *Helicoverpa armigera* (A) and *Mythimna unipuncta* (B) larvae fed on the experimental diets is shown in Figure 4, Figure 5 and Figure 6, respectively.

Neither the Bt toxin nor the vitamins added to the diet were able to modify the enzymatic activity of SOD enzyme in either species (*H. armigera*: F_140, 11_ = 1.2190; *p* = 0.2798; *M. unipuncta*: F_132, 11_ = 0.2702; *p* = 0.9902) (Figure 4A,B) or the CAT enzyme activity in *M. unipuncta* larvae (F_146, 11_ = 1.0271; *p* = 0.4256) (Figure 5B). The CAT activity in *H. armigera* larvae fed on the Bt diet was lower than the activity of larvae fed on the non-Bt diet (F_128, 1_ = 63.4029; *p* = 0.001), while the presence of added vitamins did not result in any effect (Figure 5A).

The activity of GST enzymes of the larvae of *Helicoverpa armigera* and *Mythimna unipuncta* fed on the non-Bt and Bt diet with or without vitamins are shown in Figure 6. Although the Tukey’s test indicate no differences on the LS means to the GST response to diets (Figure 6A), the ANOVA analysis indicate that larvae fed on the Bt diets showed a higher GST activity than the larvae fed on the non-Bt diets (F_147, 1_ = 5.4709; *p* = 0.02) and that this activity was modified by the presence of AsA (F_147, 2_ = 4.45.87; *p* = 0.0132) and β-carotene (F_147,2_ = 4.45.87; *p* = 0.0132). (Figure 6A).

In *M. unipuncta* the activity of GST enzymes showed double interactions between the type of diet and AsA (F_106, 2_ = 4.1450; *p* < 0.0185) as well as the type of diet and β-carotene (F_106, 2_ = 7.0815; *p* = 0.009) but the addition of the two vitamins did not modify the GST activity levels in larvae fed on the non-Bt diet (Figure 6B).

## 4. Discussion

Bt toxin analysis in the diets showed a Cry1Ab toxin concentration similar to the one that can be generally found in maize field crops [26]; the results of this work could, therefore, be extrapolated to the field situation. Larvae of *Helicoverpa armigera* fed on the Bt diet showed an extended development and a lower pupal weight compared to the ones fed on the non-Bt diet; in *Mythimna unipuncta*, the prolongation of development and the reduction in the pupal weight were affected by the presence of vitamins, indicating an effect of oxidative stress triggered by Bt toxin ingestion, as previously reported [15,29].

The biofortified crops that are currently being developed are enriched in micronutrients and vitamins, especially vitamins A and C [30]. Vitamin increase in these crops could favor the performance of phytophagous insects that grow on them in terms of a shorter development, greater pupal weight, and higher fertility of resulting adults. However, since the addition of vitamins A or C (β-carotene and ascorbic acid: AsA, respectively) to the non-Bt diet did not modify either the duration of the last instar or the weight of the resulting pupae of *H. armigera* or *M. unipuncta*, it is not likely that the populations of these phytophagous insects would increase in crops biofortified with these vitamins.

Vitamin increase in Bt crops could mitigate the effect of the toxin in the phytophagous insects that feed on these crops. Zanga et al. [13] observed that larvae of *O. nubilalis*, which are highly susceptible to Bt toxin, suffered less mortality when fed on Bt plants and diets (both containing Cry1Ac toxin) with a high content of β-carotene, in comparison to larvae fed on diets or Bt plants without β-carotene. On the contrary, Girón-Calva [14] found that neonate larvae of *O. nubilalis* fed on Bt diets supplemented with β-carotene showed higher mortality. Deeper analysis should be performed to understand the disparity between these results since the effect of vitamins could be intensified in species poorly susceptible to Bt, thus increasing their damage to the crop.

The results recorded here show that the addition of vitamins to the Bt diet affected the two studied species in a substantially different way. While the duration of last instar of *H. armigera* did not change due to the presence of vitamins, the duration of last instar of *M. unipuncta* was modified not only by β-carotene addition but also by the interaction between the two vitamins. Moreover, the interaction of vitamins produced an increase in the weight of *H. armigera* pupae but a reduction in the weight of *M. unipuncta* pupae. In no case the weight of *H. armigera* pupae fed on the Bt diet enriched with both vitamins approached to the weight of pupae developed from larvae fed on the non-Bt diet.

The lower weight of the pupae could imply smaller and less fertile adults, as pupal weight has often been correlated with female fecundity in many Lepidoptera [31,32,33,34]. Therefore, the lower performance of this phytophagous insect on Bt biofortified crops could lead to a reduction in insect pest pressure in those crops compared with non-biofortified Bt cultures.

The mortality of *M. unipuncta* larvae was not affected by vitamin presence whereas the mortality of *H. armigera* larvae fed on the Bt diet was reduced when it also contained β-carotene, as it had previously been observed in *O. nubilalis* with Cry1Ac toxin [13]. The response of *H. armigera* larvae to the toxin concentration seems to be stronger than that of *M. unipuncta* larvae, probably reflecting the higher susceptibility of *H. armigera* larvae to the Bt toxin [18]. Therefore, it does not seem that there is a unique response of phytophagous insects to biofortified Bt plants, and, consequently, vitamin enrichment in these crops would not necessarily turn out to be beneficial for pests feeding on them. *H. armigera* and *M. unipuncta* larvae fed on Bt diet showed evidence of oxidative stress, which implies an increase in reactive oxygen species (ROS) due to the imbalance of oxidants and antioxidants. Insects have a suite of enzymes dedicated to the removal of damaging ROS. Among those enzymes we can find CAT, SOD, or GST which activate or increase their expression as a response to oxidative stress [10].

On the other hand, β-carotene and AsA are non-enzymatic compounds that can either enhance or react against enzymatic antioxidants to reduce oxidative stress when insects are fed on a Bt diet. Although *H. armigera* larvae responded to the Bt toxin ingestion by reducing the expression of CAT enzymes, the vitamins added to the Bt diet did not modify the levels of SOD or CAT enzymatic activity in *H. armigera* or in *M. unipuncta,* showing differences between the two species and from what has been previously recorded [13] in *O. nubilalis*. However, GST could have an antioxidant role affecting the insect response to Bt diets in both species, as indicated by Enayati et al. [35] and Dubovskiy et al. [15], since a significant increase in GST activity was found in the body of larvae after feeding on the toxin. β-carotene, and AsA in *M. unipuncta* may also have an antioxidant effect by scavenging cell ROS since GST activity decreased in larvae fed on the Bt diet enriched with vitamins, similarly to what happens in *Lymantria dispar* larvae [36]. This result contrasts the ones obtained by Zanga et al. [13], who reported that this enzyme was not likely to play a role in *O. nubilalis* antioxidant defenses.

These results indicate that antioxidant enzymes might slightly contribute to reducing susceptibility to Bt toxin in *H. armigera* larvae and not at all in *M. unipuncta*’s ones. These results complement those obtained in *M. unipuncta* by González-Cabrera and colleagues [37]. These authors demonstrated that resistance to Bt in *M. unipuncta* was not due to photolytic degradation of the toxin or to the binding of the toxin to midgut receptors.

Our results suggest that the addition of vitamins in stacked plants does not necessarily have a beneficial effect on Lepidopteran phytophagous insects, which could lead to increased crop damage. On the contrary, it could be detrimental to pests in some cases. However, the effects of stacked biofortified and Bt plants on pest larval mortality should be studied in each Lepidopteran species. The variability observed here in the responses to the Bt toxin and the different interactions between the two vitamins in *H. armigera* and *M. unipuncta* larvae, together with the one previously reported in *O. nubilalis* [13,14], suggests that these investigations would better be performed on a case-by-case basis. The effect of both Bt toxin and vitamins on the fitness of these species indicates that the effect on their reproduction should also be studied.

## 5. Conclusions

In both *H. armigera* and *M. unipuncta* larvae, feeding on the Bt diet increased oxidative stress, manifested in the lengthening of the duration of the larval state and in the decrease in the weight of the pupae. The addition of AsA and β-carotene to the Bt diet did not mitigate the oxidative stress in *M. unipuncta larvae*. However, *H. armigera* larvae fed on the Bt diet enriched with both vitamins showed lower mortality. It does not appear that CAT or SOD enzymes contribute to the low susceptibility of *M. unipuncta* or *H. armigera* larvae to the Bt toxin, but GST enzymes might contribute to it to a small extent. The addition of β-carotene reduced the activity of GST enzymes in *M. unipuncta* larvae.

Biofortified Bt cultures do not necessarily increase the damage caused by Lepidoptera, so it would be necessary to study their effect species by species, considering other aspects of insect biology as well.

## Figures and Tables

**Table 1:** Nomenclature used in the manuscript for each component. The number of components in each diet is shown as % of total weight.

Diets			% Lyophilized Leaves		% AsA	% β-Carotene
Maize Leaves	Ascorbic Acid (AsA)	β-Carotene (β)	Non-Bt	Bt		
non-Bt	0AsA	0β	11.1	0.0	0.0	0.0
non-Bt	0AsA	0.6β	11.1	0.0	0.0	0.6
non-Bt	0.5AsA	0β	11.1	0.0	0.5	0.0
non-Bt	0.5AsA	0.6β	11.1	0.0	0.5	0.6
non-Bt	1AsA	0β	11.1	0.0	1.0	0.0
non-Bt	1AsA	0.6β	11.1	0.0	1.0	0.6
Bt	0AsA	0β	0.0	11.1	0.0	0.0
Bt	0AsA	0.6β	0.0	11.1	0.0	0.6
Bt	0.5AsA	0β	0.0	11.1	0.5	0.0
Bt	0.5AsA	0.6β	0.0	11.1	0.5	0.6
Bt	1AsA	0β	0.0	11.1	1.0	0.0
Bt	1AsA	0.6β	0.0	11.1	1.0	0.6

non-Bt is lyophilized leaves of non-Bt maize; Bt is lyophilized leaves of Bt maize; ASA is ascorbic acid, β is β-carotene.

**Table 2:** Larval mortality (%) of *Helicoverpa armigera* and *Mythimna unipuncta* fed on the different experimental diets made with two varieties of lyophilized maize leaves (non-Bt or Bt), three AsA concentrations (0, 0.5, 1% of diet w) and two β-carotene ones (0, 0.6% w).

Diet	Ascorbic Acid (AsA)	β-Carotene	Mortality (%)	
			*H. armigera*	*M. unipuncta*
non-Bt	0	0	6	3
non-Bt	0	0.6	6	0
non-Bt	0.5	0	4	0
non-Bt	0.5	0.6	3	3
non-Bt	1	0	6	0
non-Bt	1	0.6	13	0
Bt	0	0	70	14
Bt	0	0.6	43	6
Bt	0.5	0	50	0
Bt	0.5	0.6	45	9
Bt	1	0	55	0
Bt	1	0.6	3	0

## Data Availability

The data presented in this study are available in article and Supplementary Materials.

## Supplementary Materials

The statistical analysis done and the data used for them can be found in the manuscript-supplementary:pdf. The following are available online at https://www.mdpi.com/article/10.3390/insects12080718/s1.
