# Supplementary material for: Fitness Costs of Two Maize Lepidopteran Pests Fed on Bacillus thuringiensis (Bt) Diets Enriched with Vitamins A and C
Source: Insects. 2021 Aug 11;12(8):718. doi: 10.3390/insects12080718 (PMC8396949; doi:10.3390/insects12080718)
Supplement: Supplementary file 1 [file insects-12-00718-s001.zip › insects-1275377-supplementary.pdf]

Helicoverpa armigera

L6 DURATION

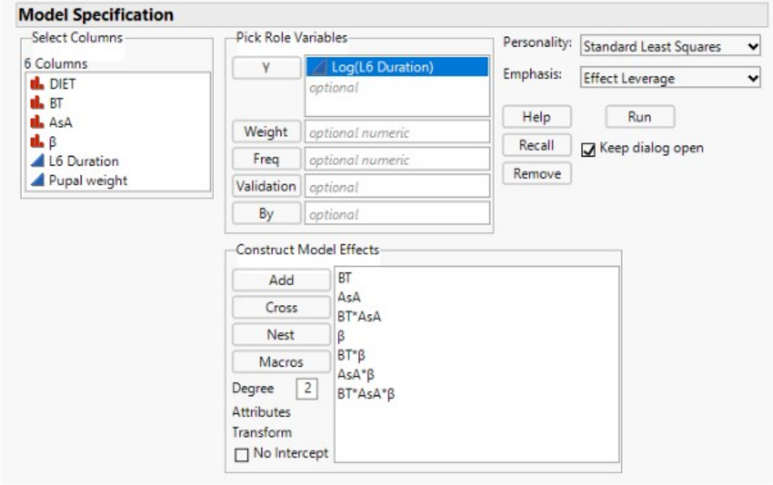

Analysis of Variance

Source	DF	Sum of Squares	Mean Square	F Ratio
Model	11	4.6075681	0.418870	33.2537
Error	254	3.1994284	0.012596	Prob > F
C. Total	265	7.8069965		<.0001*

Effect Tests

Source	Nparm	DF	Sum of Squares	F Ratio	Prob > F
BT	1	1	2.7073508	214.9344	<.0001*
AsA	2	2	0.1616680	6.4173	0.0019*
BT*AsA	2	2	0.0378784	1.5036	0.2243
β	1	1	0.0007077	0.0562	0.8128
BT*β	1	1	0.0026317	0.2089	0.6480
AsA*β	2	2	0.0537390	2.1331	0.1206
BT*AsA*β	2	2	0.0284395	1.1289	0.3250

LSMeans Differences Tukey HSD

Differences are on transformed Y's

α= 0.050 Q= 3.29887

Level					Least Sq Mean
Bt,1,0.6	A				9.7987005
Bt,1,0	A	B			9.4079445
Bt,0,0	A	B			9.1215872
Bt,0.5,0	A	B	C		8.9553899
Bt,0.5,0.6	A	B			8.9134782
Bt,0,0.6		B			8.6995721
No,1,0.6			C	D	7.5380538
No,0,0.6				D	7.3387721
No,0,0				D E	7.0975726
No,1,0				D E	7.0974213
No,0.5,0				D E	6.9876394
No,0.5,0.6				E	6.5952056

Levels not connected by same letter are significantly different.

LSMeans Differences Student's t

Differences are on transformed Y's

α= 0.050 t= 1.96935

Level			Least Sq Mean
Bt	A		9.1424068
No		B	7.1030027

Levels not connected by same letter are significantly different.

LSMeans Differences Student's t

Differences are on transformed Y's

α= 0.050 t= 1.96935

Level			Least Sq Mean
1	A		8.3802266
0		B	8.0181723
0.5		B	7.7879406

Levels not connected by same letter are significantly different.

LSMeans Differences Tukey HSD

Differences are on transformed Y's

α= 0.050 Q= 2.3576

Level			Least Sq Mean
1	A		8.3802266
0	A	B	8.0181723
0.5		B	7.7879406

Levels not connected by same letter are significantly different.

Mythimna unipuncta

L6 DURATION

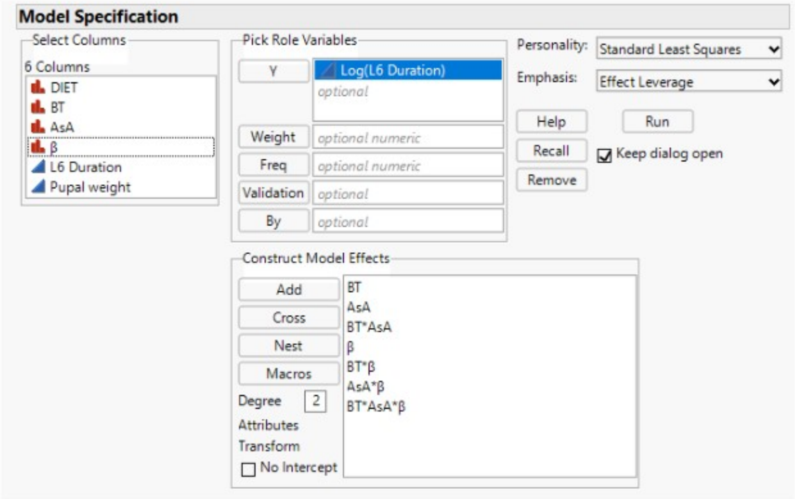

LSMeans Differences Tukey HSD

Differences are on transformed Y's

α= 0.050 Q= 3.29189

Level				Least Sq Mean
Bt,1.0,0.0	A			12.401790
Bt,0.5,0.6	A			12.373090
Bt,0.0,0.0	A			11.910497
Bt,0.5,0.0	A			11.777165
Bt,1.0,0.6		B			7.116501
Bt,0.0,0.6		B	C		6.937536
No,0.5,0.0			C	D	6.460791
No,0.0,0.0			C	D	6.412954
No,1.0,0.6				D	6.314835
No,0.0,0.6				D	6.281559
No,0.5,0.6				D	6.159661
No,1.0,0.0				D	6.063932

Levels not connected by same letter are significantly different.

Analysis of Variance

Source	DF	Sum of Squares	Mean Square	F Ratio
Model	11	26.816085	2.43783	242.7582
Error	328	3.293841	0.01004	**Prob > F**
C. Total	339	30.109926		<.0001*

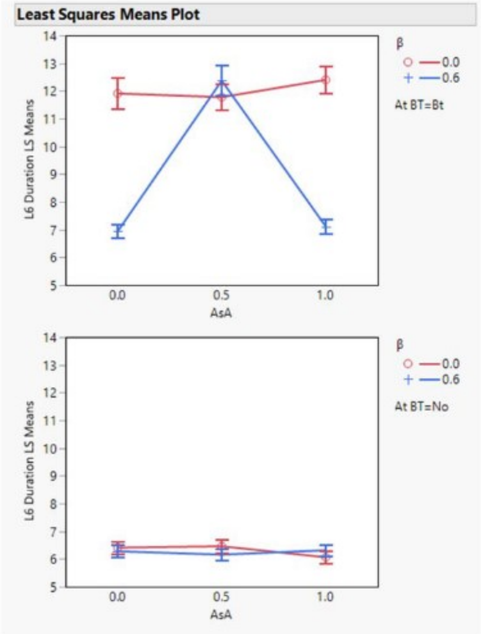

Effect Tests

Source	Nparm	DF	Sum of Squares	F Ratio	Prob > F
BT	1	1	18.473718	1839.609	<.0001*
AsA	2	2	1.308331	65.1416	<.0001*
BT*AsA	2	2	1.218599	60.6739	<.0001*
β	1	1	2.623067	261.2044	<.0001*
BT*β	1	1	2.357787	234.7879	<.0001*
AsA*β	2	2	1.273136	63.3893	<.0001*
BT*AsA*β	2	2	1.908295	95.0138	<.0001*

Helicoverpa armigera

Pupal weight

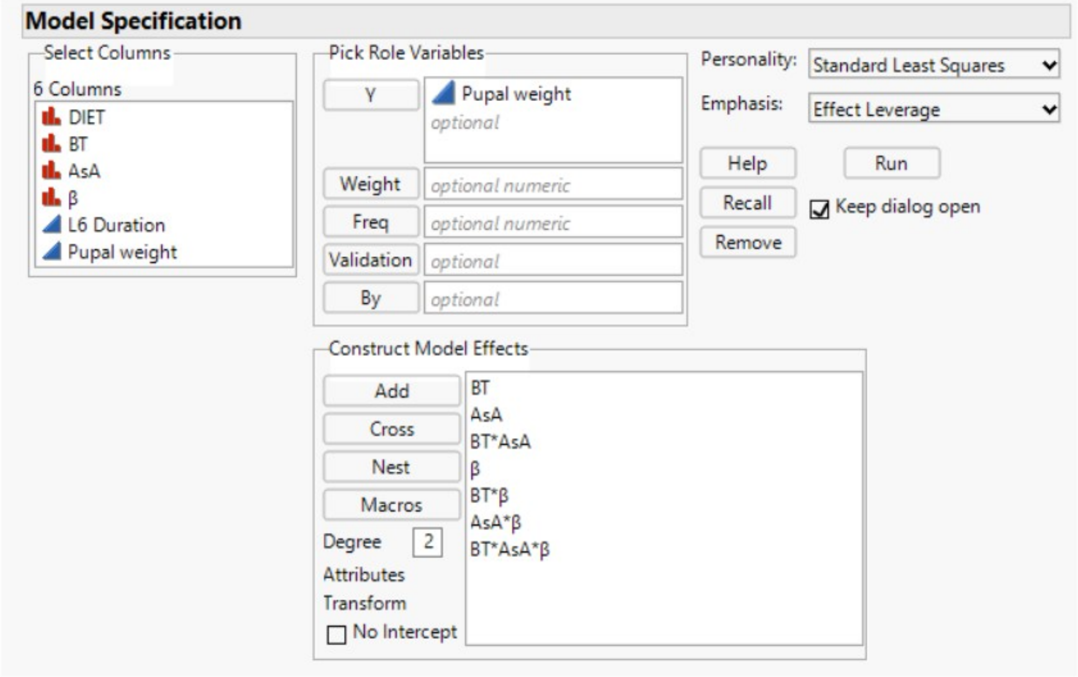

Analysis of Variance

Source	DF	Sum of Squares	Mean Square	F Ratio
Model	11	0.8395261	0.076321	50.9563
Error	253	0.3789346	0.001498	Prob > F
C. Total	264	1.2184607		<.0001*

Effect Tests

Source	Nparm	DF	Sum of Squares	F Ratio	Prob > F
BT	1	1	0.76558171	511.1494	<.0001*
AsA	2	2	0.00823619	2.7495	0.0659
BT*AsA	2	2	0.00694969	2.3200	0.1004
β	1	1	0.02050449	13.6901	0.0003*
BT*β	1	1	0.02702707	18.0449	<.0001*
AsA*β	2	2	0.00910399	3.0392	0.0496*
BT*AsA*β	2	2	0.01828768	6.1050	0.0026*

LSMeans Differences Tukey HSD

α= 0.050 Q= 3.299

Level					Least Sq Mean
No,1,0	A				0.29295769
No,0.5,0.6	A				0.28791250
No,1,0.6	A				0.28660000
No,0,0	A				0.28208000
No,0.5,0	A				0.28098306
No,0,0.6	A				0.27174194
Bt,1,0.6		B			0.20102813
Bt,0,0.6		B	C		0.18804762
Bt,0.5,0			C	D	0.13321400
Bt,0.5,0.6				D	0.12991473
Bt,0,0				D	0.12477167
Bt,1,0				D	0.11933933

Levels not connected by same letter are significantly different.

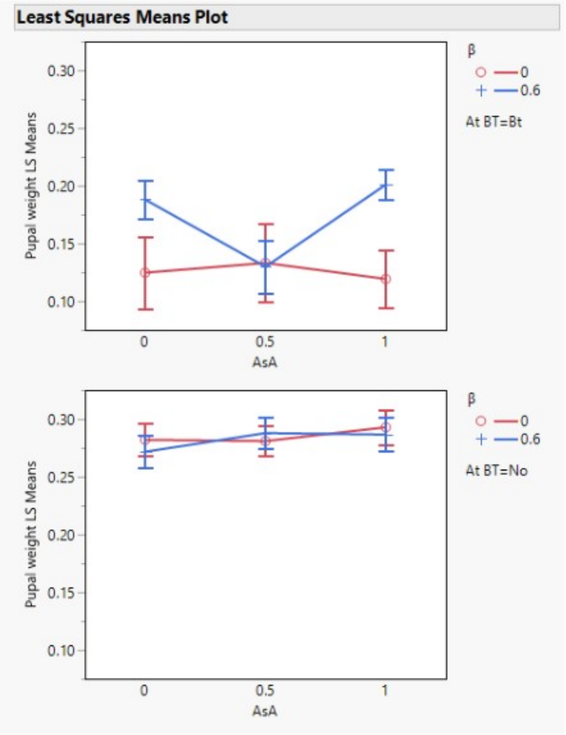

Mythimna unipuncta

PUPAL WEIGHT

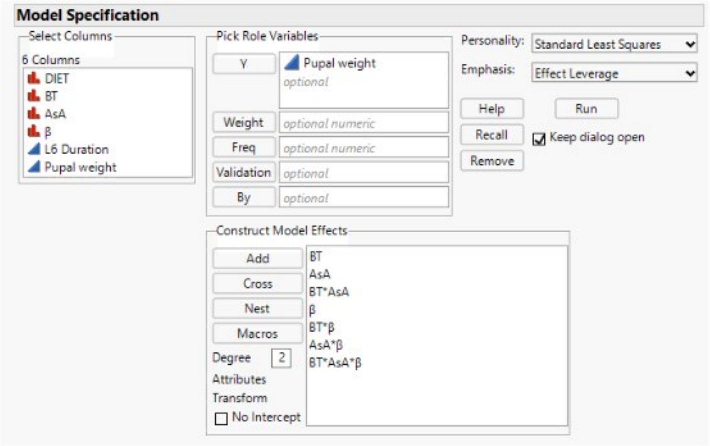

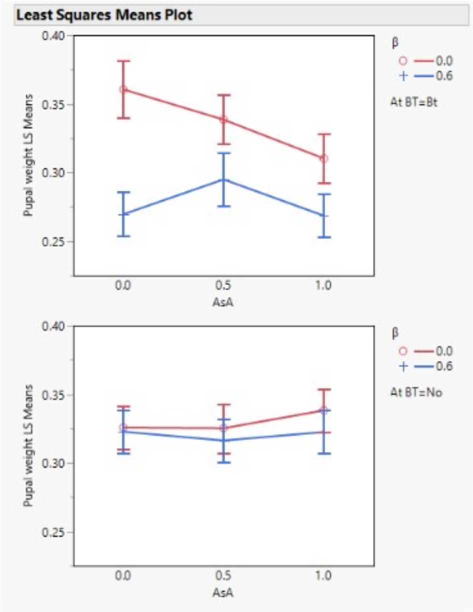

Analysis of Variance

Source	DF	Sum of Squares	Mean Square	F Ratio
Model	11	0.22109676	0.020100	9.7784
Error	328	0.67420890	0.002056	Prob > F
C. Total	339	0.89530565		<.0001*

Effect Tests

Source	Nparm	DF	Sum of Squares	F Ratio	Prob > F
BT	1	1	0.02672278	13.0005	0.0004*
AsA	2	2	0.00673473	1.6382	0.1959
BT*AsA	2	2	0.02286678	5.5623	0.0042*
β	1	1	0.09441930	45.9346	<.0001*
BT*β	1	1	0.05007920	24.3633	<.0001*
AsA*β	2	2	0.00689160	1.6764	0.1887
BT*AsA*β	2	2	0.01543681	3.7550	0.0244*

LSMeans Differences Tukey HSD

α= 0.050 Q= 3.29189

Level					Least Sq Mean
Bt,0.0,0.0	A				0.36051711
Bt,0.5,0.0	A	B	C		0.33849312
No,1.0,0.0	A	B			0.33822121
No,0.0,0.0	A	B	C		0.32577813
No,0.5,0.0	A	B	C		0.32522880
No,0.0,0.6	A	B	C		0.32272121
No,1.0,0.6	A	B	C		0.32250303
No,0.5,0.6		B	C		0.31630938
Bt,1.0,0.0		B	C		0.31022348
Bt,0.5,0.6			C	D	0.29496700
Bt,0.0,0.6				D	0.26959032
Bt,1.0,0.6				D	0.26852500

Levels not connected by same letter are significantly different.

Helicoverpa armigera

SOD

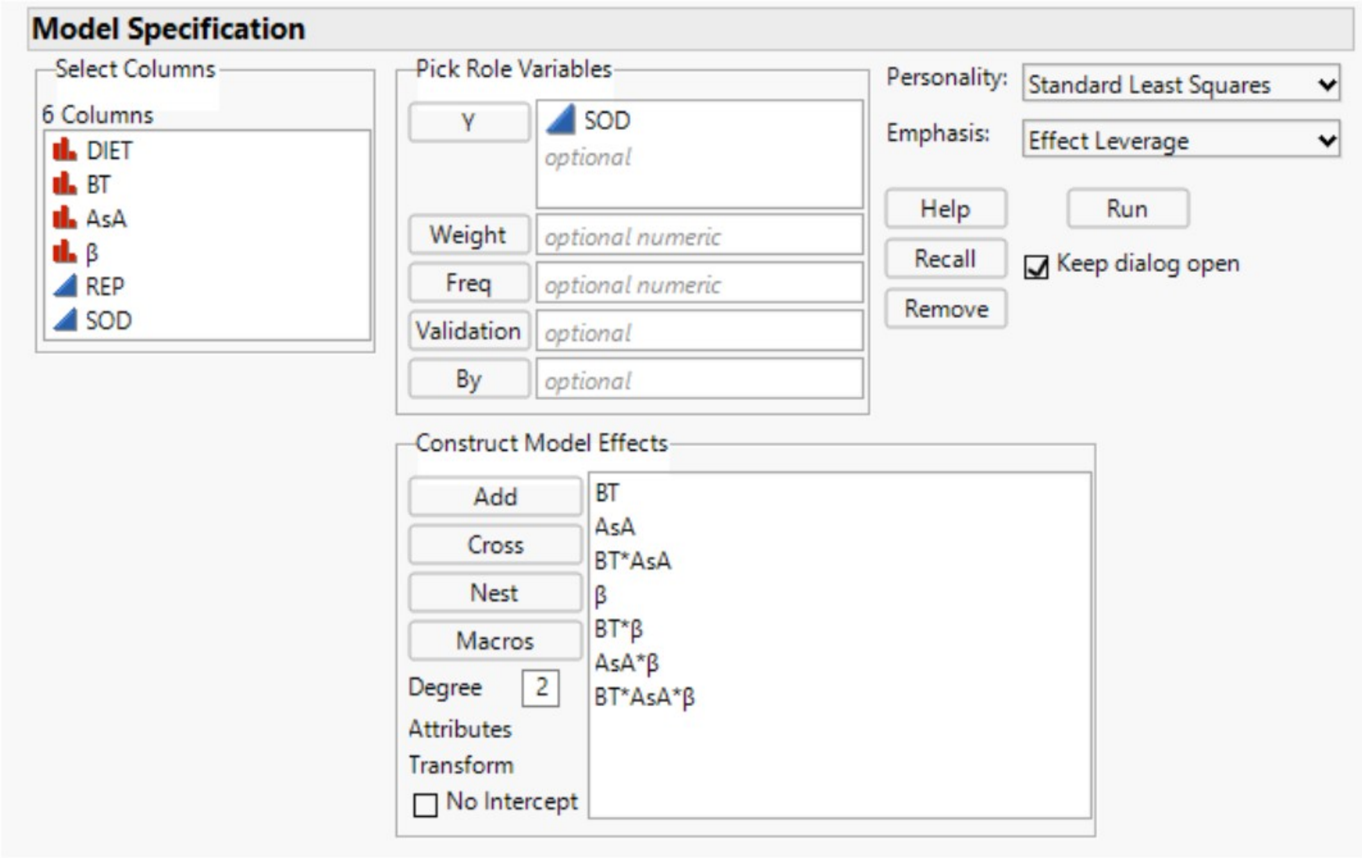

Effect Tests

Source	Nparm	DF	Sum of Squares	F Ratio	Prob > F
BT	1	1	38.77922	0.7546	0.3865
AsA	2	2	112.42319	1.0939	0.3378
BT*AsA	2	2	94.79501	0.9223	0.4000
β	1	1	19.47955	0.3791	0.5391
BT*β	1	1	0.85721	0.0167	0.8974
AsA*β	2	2	103.24199	1.0045	0.3688
BT*AsA*β	2	2	225.48053	2.1939	0.1153

Analysis of Variance

Source	DF	Sum of Squares	Mean Square	F Ratio
Model	11	689.0884	62.6444	1.2190
Error	140	7194.3543	51.3882	**Prob > F**
C. Total	151	7883.4426		0.2798

LSMeans Differences Tukey HSD

α= 0.050 Q= 3.32417

Level		Least Sq Mean
No,0,0.6	A	24.280286
No,0.5,0	A	20.216679
Bt,0.5,0.6	A	19.514167
No,1,0	A	18.220714
Bt,0,0	A	18.108889
Bt,1,0.6	A	18.085000
No,0.5,0.6	A	18.010723
No,0,0	A	17.860714
Bt,0,0.6	A	17.779643
Bt,1,0	A	17.472222
Bt,0.5,0	A	17.091389
No,1,0.6	A	15.774857

Levels not connected by same letter are significantly different.

Mythimna unipuncta

Sod

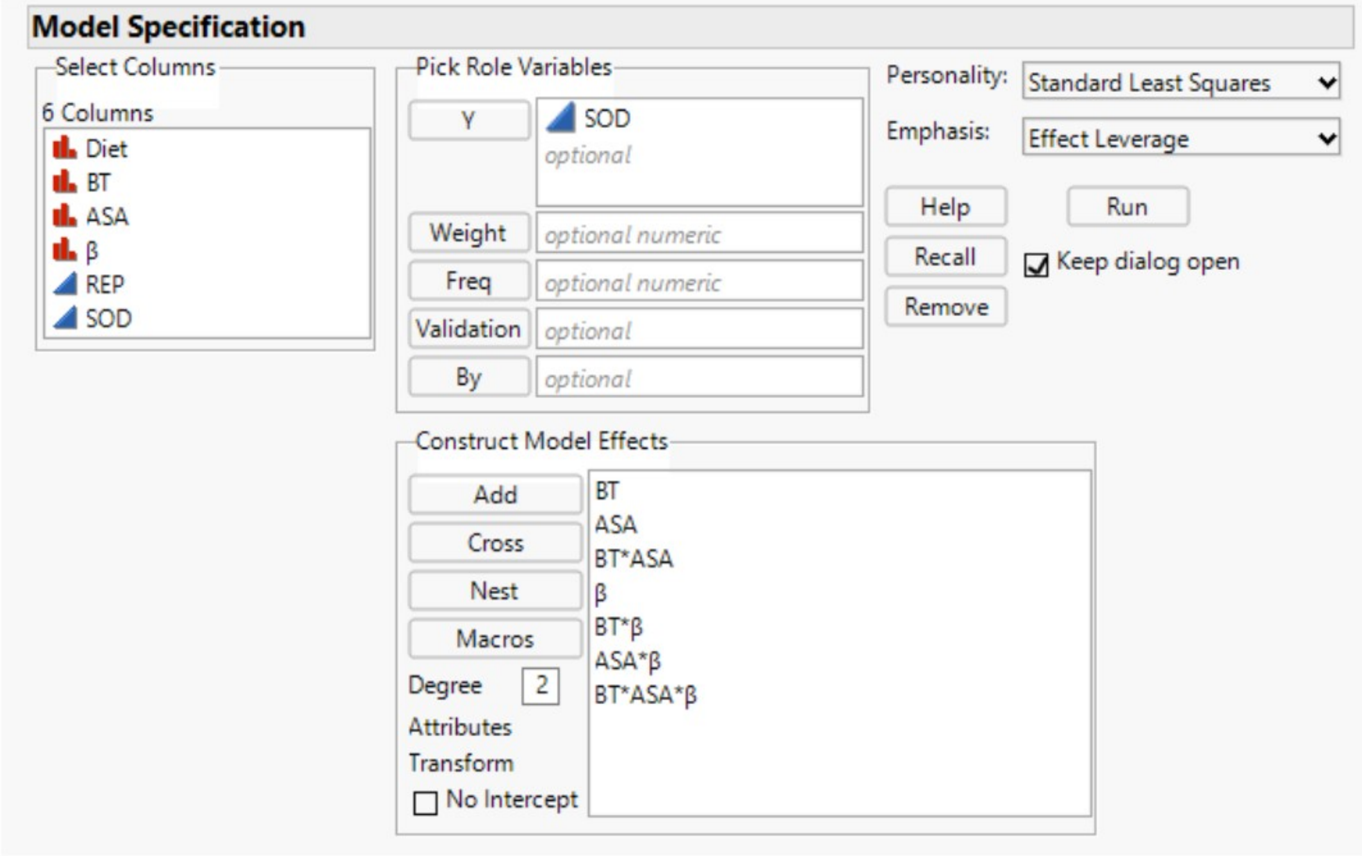

Analysis of Variance

Source	DF	Sum of Squares	Mean Square	F Ratio
Model	11	3.88594	0.35327	0.2702
Error	132	172.58344	1.30745	**Prob > F**
C. Total	143	176.46939		0.9902

Effect Tests

Source	Nparm	DF	Sum of Squares	F Ratio	Prob > F
BT	1	1	1.0958252	0.8381	0.3616
ASA	2	2	0.1844221	0.0705	0.9319
BT*ASA	2	2	0.2206431	0.0844	0.9191
β	1	1	0.2954422	0.2260	0.6353
BT*β	1	1	0.0423728	0.0324	0.8574
ASA*β	2	2	1.4045848	0.5371	0.5857
BT*ASA*β	2	2	0.7048925	0.2696	0.7641

LSMeans Differences Tukey HSD

α= 0.050 Q= 3.32759

Level		Least Sq Mean
No,1,0.6	A	3.6162500
Bt,1,0.6	A	3.5278333
No,0,0.6	A	3.5237500
No,0.5,0	A	3.3646512
No,1,0	A	3.3619167
No,0,0	A	3.3426480
Bt,0.5,0	A	3.3132246
No,0.5,0.6	A	3.3123333
Bt,0,0	A	3.3009808
Bt,0.5,0.6	A	3.2310918
Bt,0,0.6	A	3.0530000
Bt,1,0	A	3.0250751

Levels not connected by same letter are significantly different.

Helicoverpa armigera

CAT

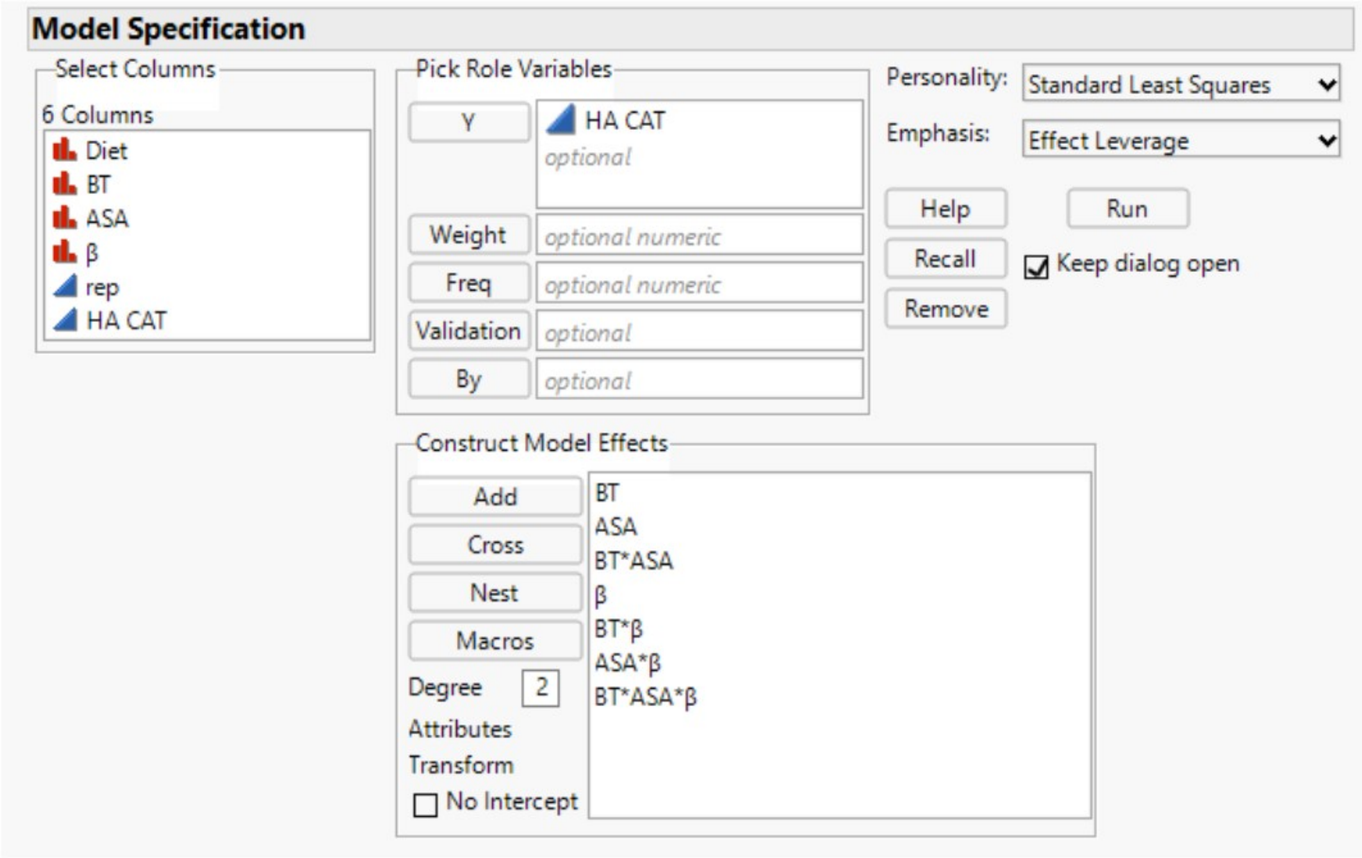

Analysis of Variance

Source	DF	Sum of Squares	Mean Square	F Ratio
Model	11	6200.021	563.638	6.3078
Error	128	11437.459	89.355	Prob > F
C. Total	139	17637.480		<.0001*

LSMeans Differences Student's t

α= 0.050 t= 1.97867

Level			Least Sq Mean
No	A		21.833072
Bt		B	8.812619

Levels not connected by same letter are significantly different.

LSMeans Differences Tukey HSD

α= 0.050 Q= 3.32947

Level						Least Sq Mean
No,1,0	A					24.341667
No,0.5,0.6	A	B				23.703795
No,0,0	A	B	C	D		21.820833
No,0.5,0	A	B	C			21.523139
No,1,0.6	A	B	C	D	E	20.858000
No,0,0.6	A	B	C	D	E	18.751000
Bt,0.5,0.6		B	C	D	E	10.803975
Bt,1,0.6			C	D	E	9.740700
Bt,0,0				D	E	8.702523
Bt,1,0					E	8.026645
Bt,0.5,0					E	7.863573
Bt,0,0.6					E	7.738300

Levels not connected by same letter are significantly different.

Effect Tests

Source	Nparm	DF	Sum of Squares	F Ratio	Prob > F
BT	1	1	5665.3798	63.4029	<.0001*
ASA	2	2	76.2038	0.4264	0.6538
BT*ASA	2	2	15.5146	0.0868	0.9169
β	1	1	0.4325	0.0048	0.9446
BT*β	1	1	60.3498	0.6754	0.4127
ASA*β	2	2	132.7822	0.7430	0.4777
BT*ASA*β	2	2	29.2608	0.1637	0.8491

Mythimna unipuncta

catalase

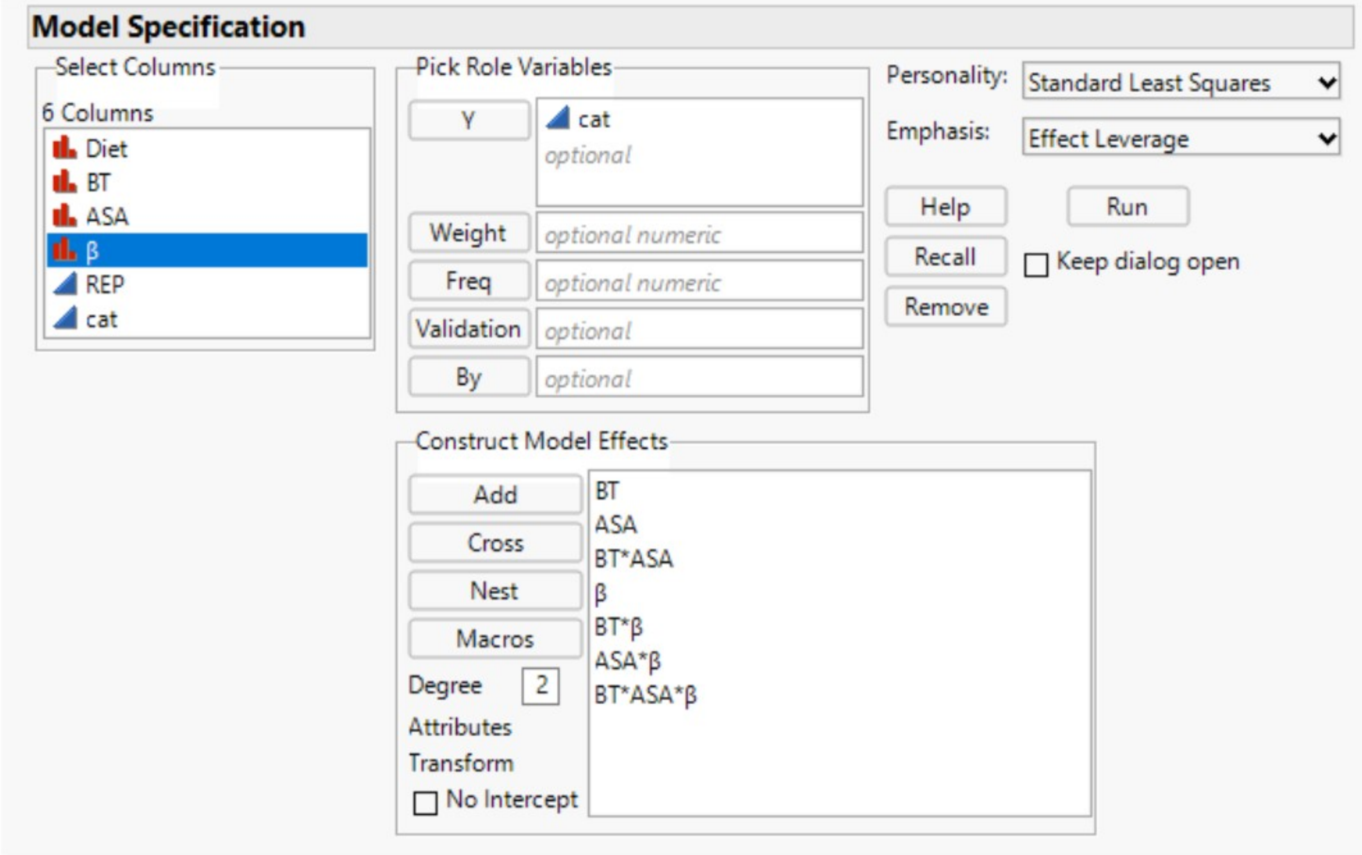

Effect Tests

Source	Nparm	DF	Sum of Squares	F Ratio	Prob > F
BT	1	1	7.479684	1.4287	0.2339
ASA	2	2	14.231792	1.3592	0.2601
BT*ASA	2	2	9.902628	0.9457	0.3908
β	1	1	2.040297	0.3897	0.5334
BT*β	1	1	0.019123	0.0037	0.9519
ASA*β	2	2	6.334414	0.6050	0.5475
BT*ASA*β	2	2	17.228265	1.6453	0.1965

Analysis of Variance

Source	DF	Sum of Squares	Mean Square	F Ratio
Model	11	59.15211	5.37746	1.0271
Error	146	764.37946	5.23548	Prob > F
C. Total	157	823.53157		0.4256

LSMeans Differences Tukey HSD

α= 0.050 Q= 3.32185

Level		Least Sq Mean
Bt,0,0.6	A	19.096429
Bt,0,0	A	18.088959
Bt,1,0	A	18.076162
Bt,0.5,0	A	17.774528
No,0,0	A	17.689437
No,1,0.6	A	17.583500
No,0.5,0	A	17.542302
Bt,0.5,0.6	A	17.503057
No,0,0.6	A	17.312857
No,1,0	A	17.292857
No,0.5,0.6	A	16.992857
Bt,1,0.6	A	16.568571

Levels not connected by same letter are significantly different.

Helicoverpa armigera

GST

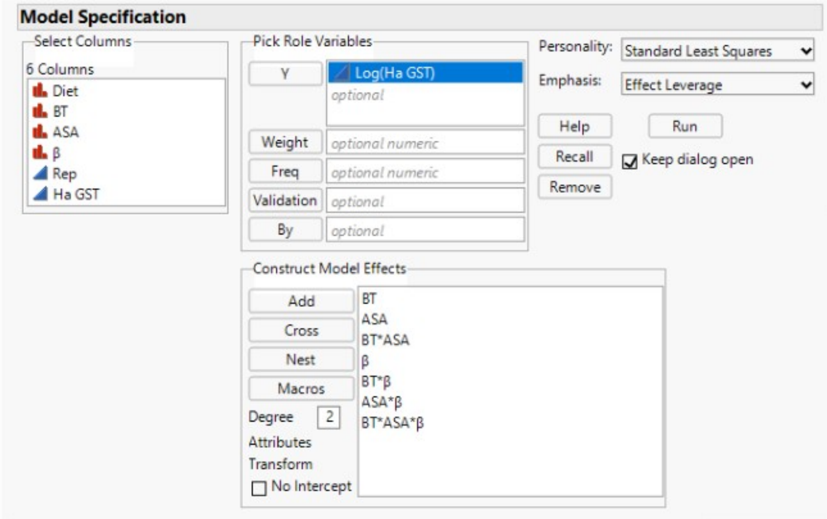

Analysis of Variance

Source	DF	Sum of Squares	Mean Square	F Ratio
Model	11	13.509196	1.22811	2.8159
Error	147	64.112189	0.43614	Prob > F
C. Total	158	77.621385		0.0023*

Effect Tests

Source	Nparm	DF	Sum of Squares	F Ratio	Prob > F
BT	1	1	2.3860475	5.4709	0.0207*
ASA	2	2	3.8892278	4.4587	0.0132*
BT*ASA	2	2	0.1195936	0.1371	0.8720
β	1	1	3.1552346	7.2345	0.0080*
BT*β	1	1	0.0052504	0.0120	0.9128
ASA*β	2	2	0.8868834	1.0167	0.3643
BT*ASA*β	2	2	1.8201885	2.0867	0.1278

LSMeans Differences Student's t

Differences are on transformed Y's

α= 0.050 t= 1.97623

Level			Least Sq Mean
Bt	A		6.0121621
No		B	4.6568879

Levels not connected by same letter are significantly different.

LSMeans Differences Tukey HSD

Differences are on transformed Y's

α= 0.050 Q= 3.32148

Level		Least Sq Mean
No,0,0	A	7.6771217
Bt,0.5,0	A	7.0513795
Bt,1,0	A	7.0357336
Bt,0,0	A	6.9289525
Bt,0,0.6	A	6.3601676
Bt,1,0.6	A	6.0995322
No,1,0	A	4.9896002
No,1,0.6	A	4.9171865
No,0,0.6	A	4.0336027
No,0.5,0	A	4.0231904
Bt,0.5,0.6	A	3.5413428
No,0.5,0.6	A	3.3367996

Levels not connected by same letter are significantly different.

LSMeans Differences Student's t

Differences are on transformed Y's

α= 0.050 t= 1.97623

Level			Least Sq Mean
0	A		6.1284075
0.6		B	4.5685547

Levels not connected by same letter are significantly different.

LSMeans Differences Tukey HSD

Differences are on transformed Y's

α= 0.050 Q= 2.36773

Level			Least Sq Mean
0	A		6.0779472
1	A	B	5.6963555
0.5		B	4.2789350

Levels not connected by same letter are significantly different.

LSMeans Differences Student's t

Differences are on transformed Y's

α= 0.050 t= 1.97623

Level				Least Sq Mean
No,0,0	A			7.6771217
Bt,0.5,0	A			7.0513795
Bt,1,0	A			7.0357336
Bt,0,0	A			6.9289525
Bt,0,0.6	A	B		6.3601676
Bt,1,0.6	A	B		6.0995322
No,1,0	A	B	C	4.9896002
No,1,0.6	A	B	C	4.9171865
No,0,0.6		B	C	4.0336027
No,0.5,0		B	C	4.0231904
Bt,0.5,0.6			C	3.5413428
No,0.5,0.6			C	3.3367996

Levels not connected by same letter are significantly different.

Mythimna unipuncta

GST

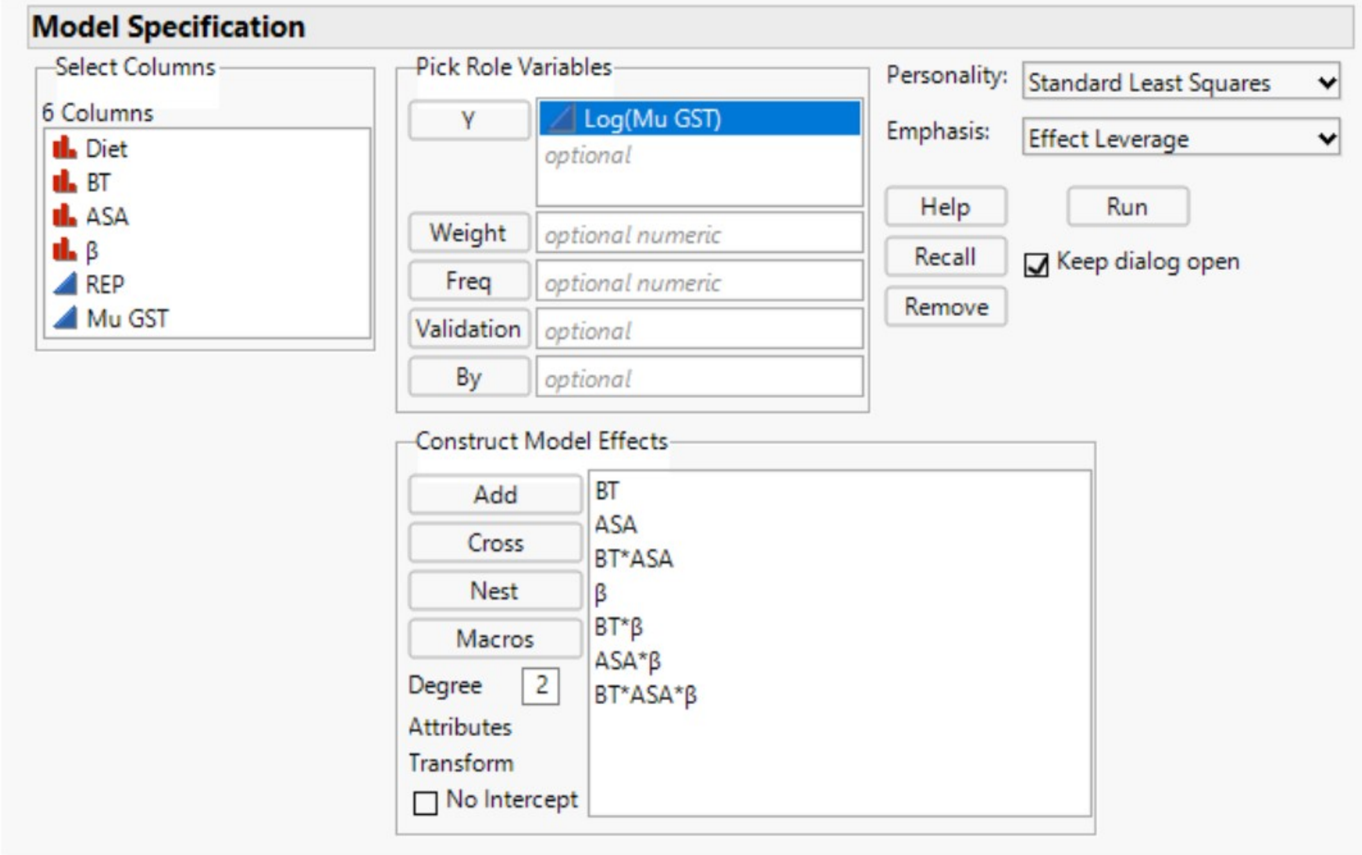

Analysis of Variance

Source	DF	Sum of Squares	Mean Square	F Ratio
Model	11	32.577805	2.96162	8.9414
Error	106	35.109728	0.33122	Prob > F
C. Total	117	67.687532		<.0001*

Effect Tests

Source	Nparm	DF	Sum of Squares	F Ratio	Prob > F
BT	1	1	23.770431	71.7655	<.0001*
ASA	2	2	4.712509	7.1138	0.0013*
BT*ASA	2	2	2.745877	4.1450	0.0185*
β	1	1	0.297443	0.8980	0.3455
BT*β	1	1	2.345566	7.0815	0.0090*
ASA*β	2	2	0.441488	0.6664	0.5157
BT*ASA*β	2	2	0.345622	0.5217	0.5950

LSMeans Differences Tukey HSD

Differences are on transformed Y's

α= 0.050 Q= 3.34233

Level						Least Sq Mean
Bt,0.5,0.0	A					40.852802
Bt,0.5,0.6	A	B				30.234258
Bt,1.0,0.0	A	B				27.098739
Bt,0.0,0.0	A	B	C			20.718303
Bt,1.0,0.6	A	B	C	D		17.303209
No,1.0,0.6		B	C	D	E	14.451965
Bt,0.0,0.6		B	C	D	E	13.537737
No,0.5,0.6			C	D	E	10.686309
No,1.0,0.0			C	D	E	9.838981
No,0.0,0.0				D	E	7.890669
No,0.5,0.0				D	E	7.871940
No,0.0,0.6					E	6.913735

Levels not connected by same letter are significantly different.

Least Squares Means Plot

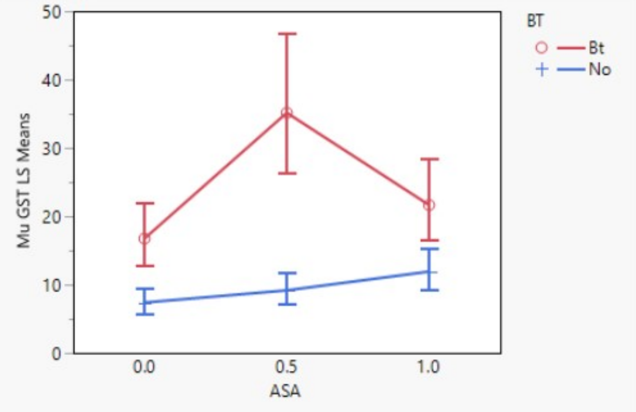

Least Squares Means Plot

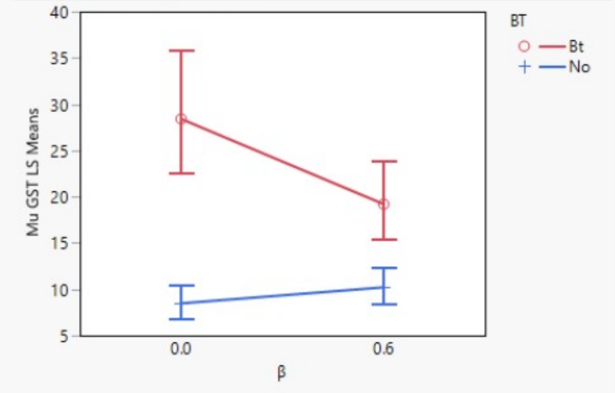

Mythimna development

DIET	BT	AsA	β	L6 Duration	Pupal weight
No0.5ASA0β	No	0.5	0.0	6	0.3125
No0.5ASA0β	No	0.5	0.0	6	0.3235
No0.5ASA0β	No	0.5	0.0	7	0.3782
No0.5ASA0β	No	0.5	0.0	6	0.3293
No0.5ASA0β	No	0.5	0.0	6	0.3216
No0.5ASA0β	No	0.5	0.0	6	0.3110
No0.5ASA0β	No	0.5	0.0	6	0.3279
No0.5ASA0β	No	0.5	0.0	7	0.3005
No0.5ASA0β	No	0.5	0.0	6	0.3312
No0.5ASA0β	No	0.5	0.0	7	0.3110
No0.5ASA0β	No	0.5	0.0	6	0.2966
No0.5ASA0β	No	0.5	0.0	6	0.3302
No0.5ASA0β	No	0.5	0.0	7	0.3350
No0.5ASA0β	No	0.5	0.0	6	0.3389
No0.5ASA0β	No	0.5	0.0	6	0.3082
No0.5ASA0β	No	0.5	0.0	6	0.3149
No0.5ASA0β	No	0.5	0.0	6	0.2986
No0.5ASA0β	No	0.5	0.0	7	0.2928
No0.5ASA0β	No	0.5	0.0	7	0.3218
No0.5ASA0β	No	0.5	0.0	7	0.2938
No0.5ASA0β	No	0.5	0.0	7	0.3584
No0.5ASA0β	No	0.5	0.0	7	0.3648
No0.5ASA0β	No	0.5	0.0	7	0.3322
No0.5ASA0β	No	0.5	0.0	7	0.3705
No0.5ASA0β	No	0.5	0.0	7	0.3274
Bt0.5ASA0β	Bt	0.5	0.0	11	0.3606
Bt0.5ASA0β	Bt	0.5	0.0	11	0.2889
Bt0.5ASA0β	Bt	0.5	0.0	11	0.3772
Bt0.5ASA0β	Bt	0.5	0.0	11	0.3308
Bt0.5ASA0β	Bt	0.5	0.0	12	0.2744
Bt0.5ASA0β	Bt	0.5	0.0	11	0.3225
Bt0.5ASA0β	Bt	0.5	0.0	11	0.3594
Bt0.5ASA0β	Bt	0.5	0.0	12	0.2635
Bt0.5ASA0β	Bt	0.5	0.0	11	0.3665
Bt0.5ASA0β	Bt	0.5	0.0	11	0.3701
Bt0.5ASA0β	Bt	0.5	0.0	11	0.3868
Bt0.5ASA0β	Bt	0.5	0.0	11	0.3653
Bt0.5ASA0β	Bt	0.5	0.0	12	0.3201
Bt0.5ASA0β	Bt	0.5	0.0	11	0.3879
Bt0.5ASA0β	Bt	0.5	0.0	12	0.3833
Bt0.5ASA0β	Bt	0.5	0.0	11	0.3356
Bt0.5ASA0β	Bt	0.5	0.0	11	0.4272
Bt0.5ASA0β	Bt	0.5	0.0	12	0.2664
Bt0.5ASA0β	Bt	0.5	0.0	13	0.3751
Bt0.5ASA0β	Bt	0.5	0.0	12	0.3958
Bt0.5ASA0β	Bt	0.5	0.0	14	0.2586
Bt0.5ASA0β	Bt	0.5	0.0	12	0.3959
Bt0.5ASA0β	Bt	0.5	0.0	13	0.3624

Bt0.5ASA0β	Bt	0.5	0.0	12	0.3262
Bt0.5ASA0β	Bt	0.5	0.0	17	0.1618
Bt0.5ASA0.6β	Bt	0.5	0.6	11	0.3261
Bt0.5ASA0.6β	Bt	0.5	0.6	11	0.2999
Bt0.5ASA0.6β	Bt	0.5	0.6	11	0.3594
Bt0.5ASA0.6β	Bt	0.5	0.6	14	0.1764
Bt0.5ASA0.6β	Bt	0.5	0.6	11	0.2856
Bt0.5ASA0.6β	Bt	0.5	0.6	12	0.3163
Bt0.5ASA0.6β	Bt	0.5	0.6	13	0.2827
Bt0.5ASA0.6β	Bt	0.5	0.6	11	0.4070
Bt0.5ASA0.6β	Bt	0.5	0.6	12	0.3231
Bt0.5ASA0.6β	Bt	0.5	0.6	11	0.4367
Bt0.5ASA0.6β	Bt	0.5	0.6	13	0.2930
Bt0.5ASA0.6β	Bt	0.5	0.6	14	0.2153
Bt0.5ASA0.6β	Bt	0.5	0.6	14	0.2205
Bt0.5ASA0.6β	Bt	0.5	0.6	12	0.3192
Bt0.5ASA0.6β	Bt	0.5	0.6	14	0.1536
Bt0.5ASA0.6β	Bt	0.5	0.6	14	0.1735
Bt0.5ASA0.6β	Bt	0.5	0.6	12	0.3112
Bt0.5ASA0.6β	Bt	0.5	0.6	12	0.3170
Bt0.5ASA0.6β	Bt	0.5	0.6	13	0.2741
Bt0.5ASA0.6β	Bt	0.5	0.6	14	0.3708
Bt0.5ASA0.6β	Bt	0.5	0.6	12	0.3331
Bt0ASA0β	Bt	0.0	0.0	11	0.3344
Bt0ASA0β	Bt	0.0	0.0	11	0.3975
Bt0ASA0β	Bt	0.0	0.0	12	0.3704
Bt0ASA0β	Bt	0.0	0.0	11	0.3284
Bt0ASA0β	Bt	0.0	0.0	13	0.2806
Bt0ASA0β	Bt	0.0	0.0	11	0.4344
Bt0ASA0β	Bt	0.0	0.0	11	0.3463
Bt0ASA0β	Bt	0.0	0.0	14	0.1911
Bt0ASA0β	Bt	0.0	0.0	11	0.3879
Bt0ASA0β	Bt	0.0	0.0	11	0.4046
Bt0ASA0β	Bt	0.0	0.0	12	0.3826
Bt0ASA0β	Bt	0.0	0.0	12	0.3890
Bt0ASA0β	Bt	0.0	0.0	13	0.3654
Bt0ASA0β	Bt	0.0	0.0	12	0.3452
Bt0ASA0β	Bt	0.0	0.0	12	0.4119
Bt0ASA0β	Bt	0.0	0.0	13	0.3945
Bt0ASA0β	Bt	0.0	0.0	13	0.3537
Bt0ASA0β	Bt	0.0	0.0	12	0.3714
Bt1ASA0β	Bt	1.0	0.0	18	0.1661
Bt1ASA0β	Bt	1.0	0.0	11	0.3511
Bt1ASA0β	Bt	1.0	0.0	11	0.3951
Bt1ASA0β	Bt	1.0	0.0	11	0.3546
Bt1ASA0β	Bt	1.0	0.0	11	0.2844
Bt1ASA0β	Bt	1.0	0.0	11	0.3249
Bt1ASA0β	Bt	1.0	0.0	12	0.2730
Bt1ASA0β	Bt	1.0	0.0	13	0.2299
Bt1ASA0β	Bt	1.0	0.0	12	0.3280

Bt1ASA0β	Bt	1.0	0.0	12	0.2892
Bt1ASA0β	Bt	1.0	0.0	11	0.4070
Bt1ASA0β	Bt	1.0	0.0	12	0.3883
Bt1ASA0β	Bt	1.0	0.0	12	0.3353
Bt1ASA0β	Bt	1.0	0.0	11	0.4070
Bt1ASA0β	Bt	1.0	0.0	12	0.2150
Bt1ASA0β	Bt	1.0	0.0	11	0.3653
Bt1ASA0β	Bt	1.0	0.0	12	0.4371
Bt1ASA0β	Bt	1.0	0.0	14	0.2668
Bt1ASA0β	Bt	1.0	0.0	13	0.3276
Bt1ASA0β	Bt	1.0	0.0	18	0.1822
Bt1ASA0β	Bt	1.0	0.0	14	0.2061
Bt1ASA0β	Bt	1.0	0.0	13	0.2315
Bt1ASA0β	Bt	1.0	0.0	13	0.2830
Bt1ASA0β	Bt	1.0	0.0	12	0.3965
Bt1ASA0β	Bt	1.0	0.0	13	0.3107
No0ASA0β	No	0.0	0.0	7	0.3100
No0ASA0β	No	0.0	0.0	7	0.3307
No0ASA0β	No	0.0	0.0	7	0.3942
No0ASA0β	No	0.0	0.0	6	0.3099
No0ASA0β	No	0.0	0.0	7	0.3301
No0ASA0β	No	0.0	0.0	6	0.3623
No0ASA0β	No	0.0	0.0	7	0.3488
No0ASA0β	No	0.0	0.0	7	0.3447
No0ASA0β	No	0.0	0.0	7	0.3061
No0ASA0β	No	0.0	0.0	6	0.3199
No0ASA0β	No	0.0	0.0	7	0.2999
No0ASA0β	No	0.0	0.0	7	0.3089
No0ASA0β	No	0.0	0.0	7	0.3528
No0ASA0β	No	0.0	0.0	6	0.3113
No0ASA0β	No	0.0	0.0	7	0.3478
No0ASA0β	No	0.0	0.0	7	0.3079
No0ASA0β	No	0.0	0.0	6	0.2872
No0ASA0β	No	0.0	0.0	7	0.3292
No0ASA0β	No	0.0	0.0	7	0.3087
No0ASA0β	No	0.0	0.0	6	0.3089
No0ASA0β	No	0.0	0.0	6	0.2743
No0ASA0β	No	0.0	0.0	7	0.2827
No0ASA0β	No	0.0	0.0	6	0.3378
No0ASA0β	No	0.0	0.0	5	0.2570
No0ASA0β	No	0.0	0.0	6	0.3402
No0ASA0β	No	0.0	0.0	6	0.3391
No0ASA0β	No	0.0	0.0	6	0.3928
No0ASA0β	No	0.0	0.0	6	0.3245
No0ASA0β	No	0.0	0.0	6	0.2990
No0ASA0β	No	0.0	0.0	6	0.2861
No0ASA0β	No	0.0	0.0	6	0.3788
No0ASA0β	No	0.0	0.0	6	0.3933
No1ASA0β	No	1.0	0.0	6	0.2965
No1ASA0β	No	1.0	0.0	6	0.3372

No1ASA0β	No	1.0	0.0	6	0.3482
No1ASA0β	No	1.0	0.0	6	0.3112
No1ASA0β	No	1.0	0.0	6	0.3722
No1ASA0β	No	1.0	0.0	6	0.3317
No1ASA0β	No	1.0	0.0	7	0.3313
No1ASA0β	No	1.0	0.0	6	0.3529
No1ASA0β	No	1.0	0.0	7	0.3440
No1ASA0β	No	1.0	0.0	6	0.3541
No1ASA0β	No	1.0	0.0	6	0.3276
No1ASA0β	No	1.0	0.0	6	0.3645
No1ASA0β	No	1.0	0.0	7	0.3555
No1ASA0β	No	1.0	0.0	6	0.3974
No1ASA0β	No	1.0	0.0	5	0.2715
No1ASA0β	No	1.0	0.0	7	0.3251
No1ASA0β	No	1.0	0.0	6	0.3164
No1ASA0β	No	1.0	0.0	6	0.3881
No1ASA0β	No	1.0	0.0	7	0.3245
No1ASA0β	No	1.0	0.0	5	0.2653
No1ASA0β	No	1.0	0.0	7	0.3478
No1ASA0β	No	1.0	0.0	5	0.2691
No1ASA0β	No	1.0	0.0	5	0.2611
No1ASA0β	No	1.0	0.0	7	0.3369
No1ASA0β	No	1.0	0.0	6	0.3781
No1ASA0β	No	1.0	0.0	6	0.3994
No1ASA0β	No	1.0	0.0	6	0.2900
No1ASA0β	No	1.0	0.0	6	0.3109
No1ASA0β	No	1.0	0.0	6	0.3633
No1ASA0β	No	1.0	0.0	6	0.3858
No1ASA0β	No	1.0	0.0	6	0.3495
No1ASA0β	No	1.0	0.0	6	0.3743
No1ASA0β	No	1.0	0.0	6	0.3799
No0ASA0.6β	No	0.0	0.6	6	0.3295
No0ASA0.6β	No	0.0	0.6	6	0.2902
No0ASA0.6β	No	0.0	0.6	6	0.2971
No0ASA0.6β	No	0.0	0.6	6	0.3659
No0ASA0.6β	No	0.0	0.6	6	0.3212
No0ASA0.6β	No	0.0	0.6	7	0.2791
No0ASA0.6β	No	0.0	0.6	7	0.3501
No0ASA0.6β	No	0.0	0.6	6	0.3259
No0ASA0.6β	No	0.0	0.6	7	0.3907
No0ASA0.6β	No	0.0	0.6	6	0.2838
No0ASA0.6β	No	0.0	0.6	6	0.3287
No0ASA0.6β	No	0.0	0.6	7	0.3337
No0ASA0.6β	No	0.0	0.6	7	0.3080
No0ASA0.6β	No	0.0	0.6	6	0.3273
No0ASA0.6β	No	0.0	0.6	6	0.2696
No0ASA0.6β	No	0.0	0.6	7	0.3285
No0ASA0.6β	No	0.0	0.6	6	0.3135
No0ASA0.6β	No	0.0	0.6	7	0.3253
No0ASA0.6β	No	0.0	0.6	7	0.3466

No0ASA0.6β	No	0.0	0.6	6	0.3160
No0ASA0.6β	No	0.0	0.6	7	0.3221
No0ASA0.6β	No	0.0	0.6	7	0.3269
No0ASA0.6β	No	0.0	0.6	6	0.3236
No0ASA0.6β	No	0.0	0.6	5	0.2713
No0ASA0.6β	No	0.0	0.6	6	0.3226
No0ASA0.6β	No	0.0	0.6	6	0.3021
No0ASA0.6β	No	0.0	0.6	7	0.3294
No0ASA0.6β	No	0.0	0.6	6	0.2978
No0ASA0.6β	No	0.0	0.6	6	0.3330
No0ASA0.6β	No	0.0	0.6	6	0.3532
No0ASA0.6β	No	0.0	0.6	6	0.3548
No0ASA0.6β	No	0.0	0.6	6	0.3171
No0ASA0.6β	No	0.0	0.6	6	0.3652
No0.5ASA0.6β	No	0.5	0.6	6	0.2666
No0.5ASA0.6β	No	0.5	0.6	6	0.2721
No0.5ASA0.6β	No	0.5	0.6	7	0.3264
No0.5ASA0.6β	No	0.5	0.6	7	0.3077
No0.5ASA0.6β	No	0.5	0.6	7	0.3237
No0.5ASA0.6β	No	0.5	0.6	5	0.2959
No0.5ASA0.6β	No	0.5	0.6	7	0.2936
No0.5ASA0.6β	No	0.5	0.6	6	0.3154
No0.5ASA0.6β	No	0.5	0.6	7	0.3802
No0.5ASA0.6β	No	0.5	0.6	5	0.2904
No0.5ASA0.6β	No	0.5	0.6	5	0.3456
No0.5ASA0.6β	No	0.5	0.6	7	0.2932
No0.5ASA0.6β	No	0.5	0.6	6	0.3384
No0.5ASA0.6β	No	0.5	0.6	6	0.3126
No0.5ASA0.6β	No	0.5	0.6	6	0.3789
No0.5ASA0.6β	No	0.5	0.6	7	0.3441
No0.5ASA0.6β	No	0.5	0.6	6	0.3126
No0.5ASA0.6β	No	0.5	0.6	6	0.2933
No0.5ASA0.6β	No	0.5	0.6	6	0.3016
No0.5ASA0.6β	No	0.5	0.6	7	0.3748
No0.5ASA0.6β	No	0.5	0.6	6	0.2934
No0.5ASA0.6β	No	0.5	0.6	6	0.2701
No0.5ASA0.6β	No	0.5	0.6	7	0.3273
No0.5ASA0.6β	No	0.5	0.6	6	0.2901
No0.5ASA0.6β	No	0.5	0.6	6	0.2254
No0.5ASA0.6β	No	0.5	0.6	6	0.3313
No0.5ASA0.6β	No	0.5	0.6	6	0.3298
No0.5ASA0.6β	No	0.5	0.6	6	0.3335
No0.5ASA0.6β	No	0.5	0.6	6	0.3287
No0.5ASA0.6β	No	0.5	0.6	6	0.3369
No0.5ASA0.6β	No	0.5	0.6	6	0.3462
No0.5ASA0.6β	No	0.5	0.6	6	0.3421
No1ASA0.6β	No	1.0	0.6	7	0.2548
No1ASA0.6β	No	1.0	0.6	7	0.3176
No1ASA0.6β	No	1.0	0.6	6	0.2646
No1ASA0.6β	No	1.0	0.6	6	0.2895

No1ASA0.6β	No	1.0	0.6	6	0.3369
No1ASA0.6β	No	1.0	0.6	6	0.4058
No1ASA0.6β	No	1.0	0.6	6	0.3554
No1ASA0.6β	No	1.0	0.6	6	0.3341
No1ASA0.6β	No	1.0	0.6	6	0.3257
No1ASA0.6β	No	1.0	0.6	6	0.3186
No1ASA0.6β	No	1.0	0.6	6	0.3565
No1ASA0.6β	No	1.0	0.6	6	0.3055
No1ASA0.6β	No	1.0	0.6	6	0.3435
No1ASA0.6β	No	1.0	0.6	6	0.3326
No1ASA0.6β	No	1.0	0.6	7	0.3506
No1ASA0.6β	No	1.0	0.6	7	0.3157
No1ASA0.6β	No	1.0	0.6	6	0.3419
No1ASA0.6β	No	1.0	0.6	5	0.2553
No1ASA0.6β	No	1.0	0.6	6	0.3534
No1ASA0.6β	No	1.0	0.6	6	0.3214
No1ASA0.6β	No	1.0	0.6	7	0.3461
No1ASA0.6β	No	1.0	0.6	6	0.3006
No1ASA0.6β	No	1.0	0.6	6	0.3286
No1ASA0.6β	No	1.0	0.6	7	0.3443
No1ASA0.6β	No	1.0	0.6	6	0.3109
No1ASA0.6β	No	1.0	0.6	10	0.2450
No1ASA0.6β	No	1.0	0.6	7	0.3232
No1ASA0.6β	No	1.0	0.6	7	0.3356
No1ASA0.6β	No	1.0	0.6	6	0.3168
No1ASA0.6β	No	1.0	0.6	7	0.3581
No1ASA0.6β	No	1.0	0.6	7	0.3215
No1ASA0.6β	No	1.0	0.6	6	0.3420
No1ASA0.6β	No	1.0	0.6	5	0.2905
Bt0ASA0.6β	Bt	0.0	0.6	7	0.2460
Bt0ASA0.6β	Bt	0.0	0.6	8	0.2388
Bt0ASA0.6β	Bt	0.0	0.6	8	0.2665
Bt0ASA0.6β	Bt	0.0	0.6	7	0.2152
Bt0ASA0.6β	Bt	0.0	0.6	7	0.3382
Bt0ASA0.6β	Bt	0.0	0.6	7	0.2613
Bt0ASA0.6β	Bt	0.0	0.6	7	0.2979
Bt0ASA0.6β	Bt	0.0	0.6	6	0.2737
Bt0ASA0.6β	Bt	0.0	0.6	6	0.2948
Bt0ASA0.6β	Bt	0.0	0.6	6	0.3009
Bt0ASA0.6β	Bt	0.0	0.6	7	0.2408
Bt0ASA0.6β	Bt	0.0	0.6	7	0.3183
Bt0ASA0.6β	Bt	0.0	0.6	6	0.2783
Bt0ASA0.6β	Bt	0.0	0.6	6	0.2445
Bt0ASA0.6β	Bt	0.0	0.6	7	0.3295
Bt0ASA0.6β	Bt	0.0	0.6	7	0.2773
Bt0ASA0.6β	Bt	0.0	0.6	6	0.2376
Bt0ASA0.6β	Bt	0.0	0.6	7	0.2239
Bt0ASA0.6β	Bt	0.0	0.6	8	0.2330
Bt0ASA0.6β	Bt	0.0	0.6	8	0.2282
Bt0ASA0.6β	Bt	0.0	0.6	7	0.2846

Bt0ASA0.6β	Bt	0.0	0.6	7	0.2959
Bt0ASA0.6β	Bt	0.0	0.6	6	0.2415
Bt0ASA0.6β	Bt	0.0	0.6	7	0.2969
Bt0ASA0.6β	Bt	0.0	0.6	7	0.2261
Bt0ASA0.6β	Bt	0.0	0.6	8	0.2190
Bt0ASA0.6β	Bt	0.0	0.6	7	0.3231
Bt0ASA0.6β	Bt	0.0	0.6	7	0.3310
Bt0ASA0.6β	Bt	0.0	0.6	7	0.2243
Bt0ASA0.6β	Bt	0.0	0.6	8	0.2114
Bt0ASA0.6β	Bt	0.0	0.6	7	0.3588
Bt1ASA0.6β	Bt	1.0	0.6	8	0.2435
Bt1ASA0.6β	Bt	1.0	0.6	7	0.2850
Bt1ASA0.6β	Bt	1.0	0.6	8	0.2137
Bt1ASA0.6β	Bt	1.0	0.6	6	0.2488
Bt1ASA0.6β	Bt	1.0	0.6	6	0.2801
Bt1ASA0.6β	Bt	1.0	0.6	7	0.2765
Bt1ASA0.6β	Bt	1.0	0.6	7	0.3170
Bt1ASA0.6β	Bt	1.0	0.6	6	0.2722
Bt1ASA0.6β	Bt	1.0	0.6	8	0.2707
Bt1ASA0.6β	Bt	1.0	0.6	8	0.2583
Bt1ASA0.6β	Bt	1.0	0.6	7	0.2645
Bt1ASA0.6β	Bt	1.0	0.6	7	0.2440
Bt1ASA0.6β	Bt	1.0	0.6	7	0.3276
Bt1ASA0.6β	Bt	1.0	0.6	8	0.3017
Bt1ASA0.6β	Bt	1.0	0.6	7	0.3037
Bt1ASA0.6β	Bt	1.0	0.6	8	0.1914
Bt1ASA0.6β	Bt	1.0	0.6	6	0.3195
Bt1ASA0.6β	Bt	1.0	0.6	7	0.2674
Bt1ASA0.6β	Bt	1.0	0.6	8	0.2608
Bt1ASA0.6β	Bt	1.0	0.6	7	0.3004
Bt1ASA0.6β	Bt	1.0	0.6	8	0.2013
Bt1ASA0.6β	Bt	1.0	0.6	7	0.2936
Bt1ASA0.6β	Bt	1.0	0.6	6	0.3268
Bt1ASA0.6β	Bt	1.0	0.6	6	0.2599
Bt1ASA0.6β	Bt	1.0	0.6	7	0.2315
Bt1ASA0.6β	Bt	1.0	0.6	8	0.2098
Bt1ASA0.6β	Bt	1.0	0.6	7	0.3006
Bt1ASA0.6β	Bt	1.0	0.6	9	0.2004
Bt1ASA0.6β	Bt	1.0	0.6	7	0.2980
Bt1ASA0.6β	Bt	1.0	0.6	7	0.2694
Bt1ASA0.6β	Bt	1.0	0.6	7	0.3081
Bt1ASA0.6β	Bt	1.0	0.6	7	0.2466

Mythimna mortality

BT	AsA	β	STATE	NUMBER
No	0	0	dead	1
No	0.5	0	dead	0
No	1	0	dead	0
No	0	0.6	dead	0
No	0.5	0.6	dead	1
No	1	0.6	dead	0
Bt	0	0.6	dead	2
Bt	0.5	0.6	dead	2
Bt	1	0.6	dead	0
Bt	0	0	dead	3
Bt	0.5	0	dead	0
Bt	1	0	dead	0
No	0	0	alive	32
No	0.5	0	alive	25
No	1	0	alive	33
No	0	0.6	alive	33
No	0.5	0.6	alive	32
No	1	0.6	alive	33
Bt	0	0.6	alive	31
Bt	0.5	0.6	alive	21
Bt	1	0.6	alive	33
Bt	0	0	alive	18
Bt	0.5	0	alive	25
Bt	1	0	alive	25

Helicoverpa development

DIET	BT	AsA	β	L6 Duration	Pupal weight
No0.5ASA0β	No	0.5	0	8	0.240729
No0.5ASA0β	No	0.5	0	7	0.289014
No0.5ASA0β	No	0.5	0	8	0.271005
No0.5ASA0β	No	0.5	0	8	0.284055
No0.5ASA0β	No	0.5	0	7	0.270396
No0.5ASA0β	No	0.5	0	7	0.283098
No0.5ASA0β	No	0.5	0	7	0.306675
No0.5ASA0β	No	0.5	0	7	0.33147
No0.5ASA0β	No	0.5	0	7	0.286578
No0.5ASA0β	No	0.5	0	7	0.284751
No0.5ASA0β	No	0.5	0	7	0.256389
No0.5ASA0β	No	0.5	0	7	0.265611
No0.5ASA0β	No	0.5	0	7	0.289275
No0.5ASA0β	No	0.5	0	7	0.272136
No0.5ASA0β	No	0.5	0	7	0.311025
No0.5ASA0β	No	0.5	0	7	0.2848
No0.5ASA0β	No	0.5	0	7	0.2263
No0.5ASA0β	No	0.5	0	7	0.212
No0.5ASA0β	No	0.5	0	7	0.3074
No0.5ASA0β	No	0.5	0	7	0.2615
No0.5ASA0β	No	0.5	0	6	0.2535
No0.5ASA0β	No	0.5	0	7	0.3058
No0.5ASA0β	No	0.5	0	7	0.2985
No0.5ASA0β	No	0.5	0	7	0.2701
No0.5ASA0β	No	0.5	0	7	0.269
No0.5ASA0β	No	0.5	0	7	0.307
No0.5ASA0β	No	0.5	0	6	0.3112
No0.5ASA0β	No	0.5	0	6	0.3475
No0.5ASA0β	No	0.5	0	7	0.2875
No0.5ASA0β	No	0.5	0	7	0.2681
No0.5ASA0β	No	0.5	0	7	0.3141
No0.5ASA0β	No	0.5	0	7	0.338
No0.5ASA0β	No	0.5	0	7	0.2322
No0.5ASA0β	No	0.5	0	7	0.2602
No0.5ASA0β	No	0.5	0	7	0.2375
Bt0.5ASA0.6β	Bt	0.5	0.6	10	0.1464
Bt0.5ASA0.6β	Bt	0.5	0.6	9	0.1278
Bt0.5ASA0.6β	Bt	0.5	0.6	8	0.1471
Bt0.5ASA0.6β	Bt	0.5	0.6	8	0.1416
Bt0.5ASA0.6β	Bt	0.5	0.6	12	0.0927
Bt0.5ASA0.6β	Bt	0.5	0.6	10	0.1109
Bt0.5ASA0.6β	Bt	0.5	0.6	9	0.1462
Bt0.5ASA0.6β	Bt	0.5	0.6	12	0.0977
Bt0.5ASA0.6β	Bt	0.5	0.6	6	0.1290
Bt0.5ASA0.6β	Bt	0.5	0.6	9	0.1889
Bt0.5ASA0.6β	Bt	0.5	0.6	7	0.1007
Bt0ASA0β	Bt	0	0	8	0.1415
Bt0ASA0β	Bt	0	0	10	0.1286

Bt0ASA0β	Bt	0	0	10	0.1130
Bt0ASA0β	Bt	0	0	10	0.1501
Bt0ASA0β	Bt	0	0	8	0.1060
Bt0ASA0β	Bt	0	0	9	0.1096
Bt0.5ASA0β	Bt	0.5	0	8	0.1410
Bt0.5ASA0β	Bt	0.5	0	10	0.1509
Bt0.5ASA0β	Bt	0.5	0	10	0.1155
Bt0.5ASA0β	Bt	0.5	0	9	0.1122
Bt0.5ASA0β	Bt	0.5	0	8	0.1465
Bt1ASA0β	Bt	1.0	0	10	0.0949
Bt1ASA0β	Bt	1.0	0	9	0.0914
Bt1ASA0β	Bt	1.0	0	9	0.0908
Bt1ASA0β	Bt	1.0	0	10	0.1368
Bt1ASA0β	Bt	1.0	0	11	0.1023
Bt1ASA0β	Bt	1.0	0	9	0.1575
Bt1ASA0β	Bt	1.0	0	8	0.1271
Bt1ASA0β	Bt	1.0	0	9	0.1341
Bt1ASA0β	Bt	1.0	0	10	0.1391
No0ASA0β	No	0	0	7	0.3034
No0ASA0β	No	0	0	7	0.2591
No0ASA0β	No	0	0	7	0.2525
No0ASA0β	No	0	0	7	0.2751
No0ASA0β	No	0	0	7	0.2903
No0ASA0β	No	0	0	8	0.2779
No0ASA0β	No	0	0	7	0.3051
No0ASA0β	No	0	0	8	0.3430
No0ASA0β	No	0	0	8	0.2913
No0ASA0β	No	0	0	7	0.3014
No0ASA0β	No	0	0	7	0.2951
No0ASA0β	No	0	0	7	0.2901
No0ASA0β	No	0	0	7	0.2930
No0ASA0β	No	0	0	9	0.2775
No0ASA0β	No	0	0	6	0.2668
No0ASA0β	No	0	0	6	0.2901
No0ASA0β	No	0	0	8	0.2912
No0ASA0β	No	0	0	6	0.3456
No0ASA0β	No	0	0	7	0.3201
No0ASA0β	No	0	0	8	0.3787
No0ASA0β	No	0	0	7	0.2695
No0ASA0β	No	0	0	7	0.2915
No0ASA0β	No	0	0	7	0.2373
No0ASA0β	No	0	0	6	0.2050
No0ASA0β	No	0	0	8	0.2692
No0ASA0β	No	0	0	6	0.2198
No0ASA0β	No	0	0	7	0.2456
No0ASA0β	No	0	0	7	0.2323
No0ASA0β	No	0	0	7	0.3596
No0ASA0β	No	0	0	8	0.1853
No1ASA0β	No	1.0	0	7	0.2631
No1ASA0β	No	1.0	0	7	0.2473

No1ASA0β	No	1.0	0	8	0.2461
No1ASA0β	No	1.0	0	7	0.2327
No1ASA0β	No	1.0	0	7	0.2867
No1ASA0β	No	1.0	0	7	0.2946
No1ASA0β	No	1.0	0	7	0.3067
No1ASA0β	No	1.0	0	7	0.3345
No1ASA0β	No	1.0	0	7	0.2910
No1ASA0β	No	1.0	0	8	0.2944
No1ASA0β	No	1.0	0	7	0.3137
No1ASA0β	No	1.0	0	7	0.2972
No1ASA0β	No	1.0	0	7	0.2688
No1ASA0β	No	1.0	0	8	0.3549
No1ASA0β	No	1.0	0	8	0.3321
No1ASA0β	No	1.0	0	6	0.2677
No1ASA0β	No	1.0	0	7	0.3457
No1ASA0β	No	1.0	0	6	0.3201
No1ASA0β	No	1.0	0	7	0.2730
No1ASA0β	No	1.0	0	7	0.2899
No1ASA0β	No	1.0	0	7	0.2967
No1ASA0β	No	1.0	0	7	0.2904
No1ASA0β	No	1.0	0	7	0.2811
No1ASA0β	No	1.0	0	7	0.2510
No1ASA0β	No	1.0	0	7	0.3309
No1ASA0β	No	1.0	0	8	0.3066
No0ASA0.6β	No	0	0.6	7	0.2572
No0ASA0.6β	No	0	0.6	7	0.2733
No0ASA0.6β	No	0	0.6	7	0.2641
No0ASA0.6β	No	0	0.6	7	0.2332
No0ASA0.6β	No	0	0.6	8	0.3004
No0ASA0.6β	No	0	0.6	7	0.2353
No0ASA0.6β	No	0	0.6	7	0.2677
No0ASA0.6β	No	0	0.6	7	0.2836
No0ASA0.6β	No	0	0.6	7	0.2352
No0ASA0.6β	No	0	0.6	7	0.2904
No0ASA0.6β	No	0	0.6	10	0.2653
No0ASA0.6β	No	0	0.6	8	0.2725
No0ASA0.6β	No	0	0.6	8	0.2441
No0ASA0.6β	No	0	0.6	8	0.2834
No0ASA0.6β	No	0	0.6	7	0.3158
No0ASA0.6β	No	0	0.6	6	0.2409
No0ASA0.6β	No	0	0.6	7	0.3138
No0ASA0.6β	No	0	0.6	9	0.2927
No0ASA0.6β	No	0	0.6	9	0.3523
No0ASA0.6β	No	0	0.6	6	0.3351
No0ASA0.6β	No	0	0.6	8	0.3774
No0ASA0.6β	No	0	0.6	7	0.2053
No0ASA0.6β	No	0	0.6	6	0.2096
No0ASA0.6β	No	0	0.6	7	0.3002
No0ASA0.6β	No	0	0.6	7	0.2855
No0ASA0.6β	No	0	0.6	8	0.2859

No0ASA0.6β	No	0	0.6	7	0.2582
No0ASA0.6β	No	0	0.6	7	0.2432
No0ASA0.6β	No	0	0.6	8	0.2242
No0ASA0.6β	No	0	0.6	7	0.2586
No0ASA0.6β	No	0	0.6	8	0.2196
No1ASA0.6β	No	1.0	0.6	7	0.2708
No1ASA0.6β	No	1.0	0.6	7	0.2315
No1ASA0.6β	No	1.0	0.6	8	0.3591
No1ASA0.6β	No	1.0	0.6	9	
No1ASA0.6β	No	1.0	0.6	8	0.3000
No1ASA0.6β	No	1.0	0.6	7	0.2704
No1ASA0.6β	No	1.0	0.6	7	0.3135
No1ASA0.6β	No	1.0	0.6	7	0.2886
No1ASA0.6β	No	1.0	0.6	9	0.3373
No1ASA0.6β	No	1.0	0.6	9	0.3495
No1ASA0.6β	No	1.0	0.6	8	0.3303
No1ASA0.6β	No	1.0	0.6	8	0.3337
No1ASA0.6β	No	1.0	0.6	9	0.3055
No1ASA0.6β	No	1.0	0.6	8	0.2912
No1ASA0.6β	No	1.0	0.6	8	0.3466
No1ASA0.6β	No	1.0	0.6	8	0.3017
No1ASA0.6β	No	1.0	0.6	7	0.2517
No1ASA0.6β	No	1.0	0.6	7	0.3023
No1ASA0.6β	No	1.0	0.6	7	0.2764
No1ASA0.6β	No	1.0	0.6	7	0.3596
No1ASA0.6β	No	1.0	0.6	7	0.2190
No1ASA0.6β	No	1.0	0.6	7	0.2986
No1ASA0.6β	No	1.0	0.6	7	0.2457
No1ASA0.6β	No	1.0	0.6	7	0.2100
No1ASA0.6β	No	1.0	0.6	7	0.2148
No1ASA0.6β	No	1.0	0.6	7	0.2334
No1ASA0.6β	No	1.0	0.6	7	0.2366
No1ASA0.6β	No	1.0	0.6	8	0.2604
Bt0ASA0.6β	Bt	0	0.6	8	0.2265
Bt0ASA0.6β	Bt	0	0.6	8	0.1754
Bt0ASA0.6β	Bt	0	0.6	8	0.1883
Bt0ASA0.6β	Bt	0	0.6	10	0.1767
Bt0ASA0.6β	Bt	0	0.6	9	0.2040
Bt0ASA0.6β	Bt	0	0.6	10	0.1967
Bt0ASA0.6β	Bt	0	0.6	10	0.1890
Bt0ASA0.6β	Bt	0	0.6	9	0.1509
Bt0ASA0.6β	Bt	0	0.6	9	0.1963
Bt0ASA0.6β	Bt	0	0.6	8	0.2098
Bt0ASA0.6β	Bt	0	0.6	8	0.3336
Bt0ASA0.6β	Bt	0	0.6	10	0.1689
Bt0ASA0.6β	Bt	0	0.6	10	0.1915
Bt0ASA0.6β	Bt	0	0.6	8	0.1595
Bt0ASA0.6β	Bt	0	0.6	7	0.2927
Bt0ASA0.6β	Bt	0	0.6	5	0.1685
Bt0ASA0.6β	Bt	0	0.6	9	0.1994

Bt0ASA0.6β	Bt	0	0.6	9	0.1340
Bt0ASA0.6β	Bt	0	0.6	9	0.1518
Bt0ASA0.6β	Bt	0	0.6	10	0.1152
Bt0ASA0.6β	Bt	0	0.6	11	0.1203
Bt1ASA0.6β	Bt	1.0	0.6	7	0.2731
Bt1ASA0.6β	Bt	1.0	0.6	10	0.2283
Bt1ASA0.6β	Bt	1.0	0.6	9	0.1969
Bt1ASA0.6β	Bt	1.0	0.6	9	0.2299
Bt1ASA0.6β	Bt	1.0	0.6	8	0.2056
Bt1ASA0.6β	Bt	1.0	0.6	10	0.2475
Bt1ASA0.6β	Bt	1.0	0.6	10	0.2586
Bt1ASA0.6β	Bt	1.0	0.6	11	0.1717
Bt1ASA0.6β	Bt	1.0	0.6	9	0.2526
Bt1ASA0.6β	Bt	1.0	0.6	10	0.2422
Bt1ASA0.6β	Bt	1.0	0.6	10	0.2554
Bt1ASA0.6β	Bt	1.0	0.6	10	0.2654
Bt1ASA0.6β	Bt	1.0	0.6	9	0.2717
Bt1ASA0.6β	Bt	1.0	0.6	11	0.2222
Bt1ASA0.6β	Bt	1.0	0.6	8	0.2122
Bt1ASA0.6β	Bt	1.0	0.6	11	0.2184
Bt1ASA0.6β	Bt	1.0	0.6	9	0.2173
Bt1ASA0.6β	Bt	1.0	0.6	12	0.1457
Bt1ASA0.6β	Bt	1.0	0.6	12	0.1012
Bt1ASA0.6β	Bt	1.0	0.6	9	0.1956
Bt1ASA0.6β	Bt	1.0	0.6	9	0.1923
Bt1ASA0.6β	Bt	1.0	0.6	9	0.2227
Bt1ASA0.6β	Bt	1.0	0.6	9	0.1757
Bt1ASA0.6β	Bt	1.0	0.6	10	0.2052
Bt1ASA0.6β	Bt	1.0	0.6	9	0.1635
Bt1ASA0.6β	Bt	1.0	0.6	10	0.1601
Bt1ASA0.6β	Bt	1.0	0.6	10	0.2003
Bt1ASA0.6β	Bt	1.0	0.6	10	0.1818
Bt1ASA0.6β	Bt	1.0	0.6	11	0.1363
Bt1ASA0.6β	Bt	1.0	0.6	12	0.1383
Bt1ASA0.6β	Bt	1.0	0.6	11	0.1134
Bt1ASA0.6β	Bt	1.0	0.6	12	0.1318
No0.5ASA0.6β	No	0.5	0.6	5	0.2423
No0.5ASA0.6β	No	0.5	0.6	7	0.3167
No0.5ASA0.6β	No	0.5	0.6	7	0.2692
No0.5ASA0.6β	No	0.5	0.6	6	0.2739
No0.5ASA0.6β	No	0.5	0.6	7	0.2557
No0.5ASA0.6β	No	0.5	0.6	6	0.3086
No0.5ASA0.6β	No	0.5	0.6	7	0.2690
No0.5ASA0.6β	No	0.5	0.6	7	0.3007
No0.5ASA0.6β	No	0.5	0.6	7	0.2500
No0.5ASA0.6β	No	0.5	0.6	7	0.2969
No0.5ASA0.6β	No	0.5	0.6	5	0.2587
No0.5ASA0.6β	No	0.5	0.6	7	0.2483
No0.5ASA0.6β	No	0.5	0.6	7	0.3279
No0.5ASA0.6β	No	0.5	0.6	7	0.3410

No0.5ASA0.6β	No	0.5	0.6	7	0.2590
No0.5ASA0.6β	No	0.5	0.6	6	0.2513
No0.5ASA0.6β	No	0.5	0.6	6	0.2670
No0.5ASA0.6β	No	0.5	0.6	6	0.2738
No0.5ASA0.6β	No	0.5	0.6	6	0.2609
No0.5ASA0.6β	No	0.5	0.6	7	0.2789
No0.5ASA0.6β	No	0.5	0.6	6	0.2825
No0.5ASA0.6β	No	0.5	0.6	7	0.3004
No0.5ASA0.6β	No	0.5	0.6	7	0.3204
No0.5ASA0.6β	No	0.5	0.6	7	0.3195
No0.5ASA0.6β	No	0.5	0.6	7	0.3302
No0.5ASA0.6β	No	0.5	0.6	7	0.2930
No0.5ASA0.6β	No	0.5	0.6	7	0.3177
No0.5ASA0.6β	No	0.5	0.6	7	0.2537
No0.5ASA0.6β	No	0.5	0.6	7	0.3024
No0.5ASA0.6β	No	0.5	0.6	7	0.3132
No0.5ASA0.6β	No	0.5	0.6	7	0.3175
No0.5ASA0.6β	No	0.5	0.6	6	0.3129

Helicoverpa mortality

BT	AsA	β	STATE	NUMBER
No	0	0	alive	34
No	0.5	0	alive	53
No	1	0	alive	33
No	0	0.6	alive	31
No	0.5	0.6	alive	29
No	1	0.6	alive	35
Bt	0	0.6	alive	21
Bt	0.5	0.6	alive	11
Bt	1	0.6	alive	35
Bt	0	0	alive	6
Bt	0.5	0	alive	10
Bt	1	0	alive	9
No	0	0	dead	2
No	0.5	0	dead	2
No	1	0	dead	2
No	0	0.6	dead	2
No	0.5	0.6	dead	1
No	1	0.6	dead	5
Bt	0	0.6	dead	16
Bt	0.5	0.6	dead	9
Bt	1	0.6	dead	1
Bt	0	0	dead	14
Bt	0.5	0	dead	10
Bt	1	0	dead	11

Mythimna cat

Diet	BT	ASA	β	REP	cat
Bt.5ASA0.6β	Bt	0.5	0.6	1	15.09
Bt.5ASA0.6β	Bt	0.5	0.6	2	13.49
Bt.5ASA0.6β	Bt	0.5	0.6	3	15.15
Bt.5ASA0.6β	Bt	0.5	0.6	4	16.73
Bt.5ASA0.6β	Bt	0.5	0.6	5	17.48
Bt.5ASA0.6β	Bt	0.5	0.6	6	15.71
Bt.5ASA0.6β	Bt	0.5	0.6	7	20.96
Bt.5ASA0.6β	Bt	0.5	0.6	8	17.33
Bt.5ASA0.6β	Bt	0.5	0.6	9	18.96
Bt.5ASA0.6β	Bt	0.5	0.6	10	24.13
Bt.5ASA0β	Bt	0.5	0	1	17.32
Bt.5ASA0β	Bt	0.5	0	2	14.07
Bt.5ASA0β	Bt	0.5	0	3	15.70
Bt.5ASA0β	Bt	0.5	0	4	17.47
Bt.5ASA0β	Bt	0.5	0	5	17.22
Bt.5ASA0β	Bt	0.5	0	6	17.36
Bt.5ASA0β	Bt	0.5	0	7	20.19
Bt.5ASA0β	Bt	0.5	0	8	17.89
Bt.5ASA0β	Bt	0.5	0	9	17.97
Bt.5ASA0β	Bt	0.5	0	10	22.55
Bt0ASA0.6β	Bt	0	0.6	1	17.46
Bt0ASA0.6β	Bt	0	0.6	2	17.07
Bt0ASA0.6β	Bt	0	0.6	3	17.96
Bt0ASA0.6β	Bt	0	0.6	4	17.88
Bt0ASA0.6β	Bt	0	0.6	5	16.42
Bt0ASA0.6β	Bt	0	0.6	6	16.08
Bt0ASA0.6β	Bt	0	0.6	7	23.89
Bt0ASA0.6β	Bt	0	0.6	8	23.97
Bt0ASA0.6β	Bt	0	0.6	9	19.72
Bt0ASA0.6β	Bt	0	0.6	10	19.21
Bt0ASA0.6β	Bt	0	0.6	11	19.54
Bt0ASA0.6β	Bt	0	0.6	12	19.97
Bt0ASA0.6β	Bt	0	0.6	13	19.09
Bt0ASA0.6β	Bt	0	0.6	14	19.09
Bt0ASA0β	Bt	0	0	1	15.21
Bt0ASA0β	Bt	0	0	2	13.85
Bt0ASA0β	Bt	0	0	3	16.11
Bt0ASA0β	Bt	0	0	4	17.89
Bt0ASA0β	Bt	0	0	5	19.75
Bt0ASA0β	Bt	0	0	6	17.70
Bt0ASA0β	Bt	0	0	7	23.87
Bt0ASA0β	Bt	0	0	8	14.49
Bt0ASA0β	Bt	0	0	9	18.32
Bt0ASA0β	Bt	0	0	10	23.70
Bt1ASA0.6β	Bt	1	0.6	1	17.72
Bt1ASA0.6β	Bt	1	0.6	2	18.19
Bt1ASA0.6β	Bt	1	0.6	3	16.61
Bt1ASA0.6β	Bt	1	0.6	4	16.82

Bt1ASA0.6β	Bt	1	0.6	5	17.46
Bt1ASA0.6β	Bt	1	0.6	6	15.48
Bt1ASA0.6β	Bt	1	0.6	7	14.77
Bt1ASA0.6β	Bt	1	0.6	8	14.91
Bt1ASA0.6β	Bt	1	0.6	9	15.71
Bt1ASA0.6β	Bt	1	0.6	10	15.41
Bt1ASA0.6β	Bt	1	0.6	11	16.54
Bt1ASA0.6β	Bt	1	0.6	12	15.93
Bt1ASA0.6β	Bt	1	0.6	13	18.29
Bt1ASA0.6β	Bt	1	0.6	14	18.12
Bt1ASA0β	Bt	1	0	1	15.14
Bt1ASA0β	Bt	1	0	2	14.76
Bt1ASA0β	Bt	1	0	3	16.26
Bt1ASA0β	Bt	1	0	4	17.83
Bt1ASA0β	Bt	1	0	5	21.26
Bt1ASA0β	Bt	1	0	6	16.60
Bt1ASA0β	Bt	1	0	7	16.58
Bt1ASA0β	Bt	1	0	8	16.97
Bt1ASA0β	Bt	1	0	9	22.27
Bt1ASA0β	Bt	1	0	10	23.08
No0.5ASA0β	No	0.5	0	1	16.75
No0.5ASA0β	No	0.5	0	2	14.37
No0.5ASA0β	No	0.5	0	3	17.77
No0.5ASA0β	No	0.5	0	4	15.90
No0.5ASA0β	No	0.5	0	5	17.54
No0.5ASA0β	No	0.5	0	6	18.10
No0.5ASA0β	No	0.5	0	7	20.01
No0.5ASA0β	No	0.5	0	8	16.30
No0.5ASA0β	No	0.5	0	9	20.67
No0.5ASA0β	No	0.5	0	10	18.02
No0.5ASA0.6	No	0.5	0.6	1	16.35
No0.5ASA0.6	No	0.5	0.6	2	16.07
No0.5ASA0.6	No	0.5	0.6	3	16.72
No0.5ASA0.6	No	0.5	0.6	4	16.90
No0.5ASA0.6	No	0.5	0.6	5	16.96
No0.5ASA0.6	No	0.5	0.6	6	16.46
No0.5ASA0.6	No	0.5	0.6	7	22.36
No0.5ASA0.6	No	0.5	0.6	8	20.26
No0.5ASA0.6	No	0.5	0.6	9	13.84
No0.5ASA0.6	No	0.5	0.6	10	15.44
No0.5ASA0.6	No	0.5	0.6	11	16.41
No0.5ASA0.6	No	0.5	0.6	12	16.42
No0.5ASA0.6	No	0.5	0.6	13	16.89
No0.5ASA0.6	No	0.5	0.6	14	16.82
No0ASA0.6β	No	0	0.6	1	19.84
No0ASA0.6β	No	0	0.6	2	18.89
No0ASA0.6β	No	0	0.6	3	17.53
No0ASA0.6β	No	0	0.6	4	15.71
No0ASA0.6β	No	0	0.6	5	16.75
No0ASA0.6β	No	0	0.6	6	17.50

No0ASA0.6β	No	0	0.6	7	17.42
No0ASA0.6β	No	0	0.6	8	16.47
No0ASA0.6β	No	0	0.6	9	15.48
No0ASA0.6β	No	0	0.6	10	14.97
No0ASA0.6β	No	0	0.6	11	17.74
No0ASA0.6β	No	0	0.6	12	17.33
No0ASA0.6β	No	0	0.6	13	18.53
No0ASA0.6β	No	0	0.6	14	18.22
No0ASA0β	No	0	0	1	18.61
No0ASA0β	No	0	0	2	17.88
No0ASA0β	No	0	0	3	19.02
No0ASA0β	No	0	0	4	18.22
No0ASA0β	No	0	0	5	19.05
No0ASA0β	No	0	0	6	19.84
No0ASA0β	No	0	0	7	17.24
No0ASA0β	No	0	0	8	16.27
No0ASA0β	No	0	0	9	20.34
No0ASA0β	No	0	0	10	20.10
No0ASA0β	No	0	0	11	16.86
No0ASA0β	No	0	0	12	16.89
No0ASA0β	No	0	0	13	15.81
No0ASA0β	No	0	0	14	15.26
No0ASA0β	No	0	0	1	15.02
No0ASA0β	No	0	0	2	16.97
No0ASA0β	No	0	0	3	17.24
No0ASA0β	No	0	0	4	17.86
No0ASA0β	No	0	0	5	17.96
No0ASA0β	No	0	0	6	17.59
No0ASA0β	No	0	0	7	17.28
No0ASA0β	No	0	0	8	18.30
No0ASA0β	No	0	0	9	18.23
No0ASA0β	No	0	0	10	16.72
No1ASA0.6β	No	1	0.6	1	18.50
No1ASA0.6β	No	1	0.6	2	17.87
No1ASA0.6β	No	1	0.6	3	15.73
No1ASA0.6β	No	1	0.6	4	9.90
No1ASA0.6β	No	1	0.6	5	24.50
No1ASA0.6β	No	1	0.6	6	24.15
No1ASA0.6β	No	1	0.6	7	17.18
No1ASA0.6β	No	1	0.6	8	16.75
No1ASA0.6β	No	1	0.6	9	16.81
No1ASA0.6β	No	1	0.6	10	17.32
No1ASA0.6β	No	1	0.6	11	18.42
No1ASA0.6β	No	1	0.6	12	18.41
No1ASA0.6β	No	1	0.6	13	14.82
No1ASA0.6β	No	1	0.6	14	15.81
No1ASA0β	No	1	0	1	17.48
No1ASA0β	No	1	0	2	16.85
No1ASA0β	No	1	0	3	19.31
No1ASA0β	No	1	0	4	19.21

No1ASA0β	No	1	0	5	18.86
No1ASA0β	No	1	0	6	18.05
No1ASA0β	No	1	0	7	16.21
No1ASA0β	No	1	0	8	16.69
No1ASA0β	No	1	0	9	17.73
No1ASA0β	No	1	0	10	17.29
No1ASA0β	No	1	0	11	16.22
No1ASA0β	No	1	0	12	16.63
No1ASA0β	No	1	0	13	15.58
No1ASA0β	No	1	0	14	15.99

Mythimna sod

Diet	BT	ASA	β	REP	SOD
No0ASA0β	No	0	0	1	2.85
No0ASA0β	No	0	0	2	3.50
No0ASA0β	No	0	0	3	2.26
No0ASA0β	No	0	0	4	3.54
No0ASA0β	No	0	0	5	2.85
No0ASA0β	No	0	0	6	3.40
No0ASA0β	No	0	0	7	3.73
No0ASA0β	No	0	0	8	3.32
No0ASA0β	No	0	0	9	1.51
No0ASA0β	No	0	0	10	3.86
No0ASA0β	No	0	0	11	4.17
No0ASA0β	No	0	0	12	4.17
No1ASA0β	No	1	0	1	3.01
No1ASA0β	No	1	0	2	2.88
No1ASA0β	No	1	0	3	4.93
No1ASA0β	No	1	0	4	2.71
No1ASA0β	No	1	0	5	3.29
No1ASA0β	No	1	0	6	2.93
No1ASA0β	No	1	0	7	3.20
No1ASA0β	No	1	0	8	3.07
No1ASA0β	No	1	0	9	4.07
No1ASA0β	No	1	0	10	2.76
No1ASA0β	No	1	0	11	3.95
No1ASA0β	No	1	0	12	3.56
No0ASA0.6β	No	0	0.6	1	2.25
No0ASA0.6β	No	0	0.6	2	3.21
No0ASA0.6β	No	0	0.6	3	4.32
No0ASA0.6β	No	0	0.6	4	2.63
No0ASA0.6β	No	0	0.6	5	2.89
No0ASA0.6β	No	0	0.6	6	4.51
No0ASA0.6β	No	0	0.6	7	2.05
No0ASA0.6β	No	0	0.6	8	3.54
No0ASA0.6β	No	0	0.6	9	4.78
No0ASA0.6β	No	0	0.6	10	2.89
No0ASA0.6β	No	0	0.6	11	3.56
No0ASA0.6β	No	0	0.6	12	5.66
No0.5ASA0.6	No	0.5	0.6	1	3.09
No0.5ASA0.6	No	0.5	0.6	2	3.95
No0.5ASA0.6	No	0.5	0.6	3	3.07
No0.5ASA0.6	No	0.5	0.6	4	1.97
No0.5ASA0.6	No	0.5	0.6	5	2.53
No0.5ASA0.6	No	0.5	0.6	6	4.40
No0.5ASA0.6	No	0.5	0.6	7	2.89
No0.5ASA0.6	No	0.5	0.6	8	3.81
No0.5ASA0.6	No	0.5	0.6	9	3.29
No0.5ASA0.6	No	0.5	0.6	10	2.33
No0.5ASA0.6	No	0.5	0.6	11	2.89
No0.5ASA0.6	No	0.5	0.6	12	5.51

No1ASA0.6β	No	1	0.6	1	2.33
No1ASA0.6β	No	1	0.6	2	4.96
No1ASA0.6β	No	1	0.6	3	3.52
No1ASA0.6β	No	1	0.6	4	3.44
No1ASA0.6β	No	1	0.6	5	3.81
No1ASA0.6β	No	1	0.6	6	3.09
No1ASA0.6β	No	1	0.6	7	2.43
No1ASA0.6β	No	1	0.6	8	4.35
No1ASA0.6β	No	1	0.6	9	3.34
No1ASA0.6β	No	1	0.6	10	2.82
No1ASA0.6β	No	1	0.6	11	4.69
No1ASA0.6β	No	1	0.6	12	4.63
Bt0ASA0.6β	Bt	0	0.6	1	2.10
Bt0ASA0.6β	Bt	0	0.6	2	2.48
Bt0ASA0.6β	Bt	0	0.6	3	3.20
Bt0ASA0.6β	Bt	0	0.6	4	3.81
Bt0ASA0.6β	Bt	0	0.6	5	3.21
Bt0ASA0.6β	Bt	0	0.6	6	2.99
Bt0ASA0.6β	Bt	0	0.6	7	2.00
Bt0ASA0.6β	Bt	0	0.6	8	3.62
Bt0ASA0.6β	Bt	0	0.6	9	4.22
Bt0ASA0.6β	Bt	0	0.6	10	3.02
Bt0ASA0.6β	Bt	0	0.6	11	3.48
Bt0ASA0.6β	Bt	0	0.6	12	2.51
Bt1ASA0.6β	Bt	1	0.6	1	2.21
Bt1ASA0.6β	Bt	1	0.6	2	4.22
Bt1ASA0.6β	Bt	1	0.6	3	3.73
Bt1ASA0.6β	Bt	1	0.6	4	3.30
Bt1ASA0.6β	Bt	1	0.6	5	4.14
Bt1ASA0.6β	Bt	1	0.6	6	3.48
Bt1ASA0.6β	Bt	1	0.6	7	2.79
Bt1ASA0.6β	Bt	1	0.6	8	3.09
Bt1ASA0.6β	Bt	1	0.6	9	4.14
Bt1ASA0.6β	Bt	1	0.6	10	3.40
Bt1ASA0.6β	Bt	1	0.6	11	4.75
Bt1ASA0.6β	Bt	1	0.6	12	3.09
No0ASA0β	No	0	0	1	4.79
No0ASA0β	No	0	0	2	4.79
No0ASA0β	No	0	0	3	4.79
No0ASA0β	No	0	0	4	4.79
No0ASA0β	No	0	0	5	2.47
No0ASA0β	No	0	0	6	2.46
No0ASA0β	No	0	0	7	2.35
No0ASA0β	No	0	0	8	2.74
No0ASA0β	No	0	0	9	2.87
No0ASA0β	No	0	0	10	2.37
No0.5ASA0β	No	0.5	0	1	6.38
No0.5ASA0β	No	0.5	0	2	4.79
No0.5ASA0β	No	0.5	0	3	4.79
No0.5ASA0β	No	0.5	0	4	4.79

No0.5ASA0β	No	0.5	0	5	2.04
No0.5ASA0β	No	0.5	0	6	2.15
No0.5ASA0β	No	0.5	0	7	2.08
No0.5ASA0β	No	0.5	0	8	2.16
No0.5ASA0β	No	0.5	0	9	2.38
No0.5ASA0β	No	0.5	0	10	2.09
Bt.5ASA0.6β	Bt	0.5	0.6	1	6.17
Bt.5ASA0.6β	Bt	0.5	0.6	2	4.68
Bt.5ASA0.6β	Bt	0.5	0.6	3	4.68
Bt.5ASA0.6β	Bt	0.5	0.6	4	4.68
Bt.5ASA0.6β	Bt	0.5	0.6	5	2.35
Bt.5ASA0.6β	Bt	0.5	0.6	6	1.93
Bt.5ASA0.6β	Bt	0.5	0.6	7	2.27
Bt.5ASA0.6β	Bt	0.5	0.6	8	2.05
Bt.5ASA0.6β	Bt	0.5	0.6	9	1.76
Bt.5ASA0.6β	Bt	0.5	0.6	10	1.73
Bt0ASA0β	Bt	0	0	1	4.79
Bt0ASA0β	Bt	0	0	2	4.68
Bt0ASA0β	Bt	0	0	3	4.68
Bt0ASA0β	Bt	0	0	4	4.68
Bt0ASA0β	Bt	0	0	5	2.49
Bt0ASA0β	Bt	0	0	6	2.31
Bt0ASA0β	Bt	0	0	7	2.35
Bt0ASA0β	Bt	0	0	8	2.59
Bt0ASA0β	Bt	0	0	9	2.15
Bt0ASA0β	Bt	0	0	10	2.28
Bt.5ASA0β	Bt	0.5	0	1	4.76
Bt.5ASA0β	Bt	0.5	0	2	4.68
Bt.5ASA0β	Bt	0.5	0	3	4.68
Bt.5ASA0β	Bt	0.5	0	4	4.68
Bt.5ASA0β	Bt	0.5	0	5	2.23
Bt.5ASA0β	Bt	0.5	0	6	2.12
Bt.5ASA0β	Bt	0.5	0	7	2.02
Bt.5ASA0β	Bt	0.5	0	8	4.51
Bt.5ASA0β	Bt	0.5	0	9	1.92
Bt.5ASA0β	Bt	0.5	0	10	1.53
Bt1ASA0β	Bt	1	0	1	5.11
Bt1ASA0β	Bt	1	0	2	4.68
Bt1ASA0β	Bt	1	0	3	4.68
Bt1ASA0β	Bt	1	0	4	4.68
Bt1ASA0β	Bt	1	0	5	2.33
Bt1ASA0β	Bt	1	0	6	1.94
Bt1ASA0β	Bt	1	0	7	2.40
Bt1ASA0β	Bt	1	0	8	2.34
Bt1ASA0β	Bt	1	0	9	0.00
Bt1ASA0β	Bt	1	0	10	2.08

Mythimn gst

Diet	BT	ASA	β	REP	Mu GST
No0ASA0β	No	0.0	0.0	8	2.15
No0ASA0β	No	0.0	0.0	10	2.66
No0ASA0β	No	0.0	0.0	7	5.44
No0ASA0β	No	0.0	0.0	5	5.72
No0ASA0β	No	0.0	0.0	4	6.93
No0ASA0β	No	0.0	0.0	9	9.69
No0ASA0β	No	0.0	0.0	6	11.50
No0ASA0β	No	0.0	0.0	2	17.49
No0ASA0β	No	0.0	0.0	3	18.99
No0ASA0β	No	0.0	0.0	1	20.52
No0ASA0.6β	No	0.0	0.6	9	2.00
No0ASA0.6β	No	0.0	0.6	8	2.08
No0ASA0.6β	No	0.0	0.6	6	4.67
No0ASA0.6β	No	0.0	0.6	7	4.71
No0ASA0.6β	No	0.0	0.6	10	6.51
No0ASA0.6β	No	0.0	0.6	4	10.16
No0ASA0.6β	No	0.0	0.6	2	11.34
No0ASA0.6β	No	0.0	0.6	5	11.96
No0ASA0.6β	No	0.0	0.6	3	15.16
No0ASA0.6β	No	0.0	0.6	1	20.11
No0.5ASA0β	No	0.5	0.0	5	3.09
No0.5ASA0β	No	0.5	0.0	4	3.52
No0.5ASA0β	No	0.5	0.0	6	7.79
No0.5ASA0β	No	0.5	0.0	9	8.43
No0.5ASA0β	No	0.5	0.0	7	9.00
No0.5ASA0β	No	0.5	0.0	1	10.39
No0.5ASA0β	No	0.5	0.0	2	14.78
No0.5ASA0β	No	0.5	0.0	3	14.93
No0.5ASA0.6β	No	0.5	0.6	10	4.48
No0.5ASA0.6β	No	0.5	0.6	8	5.05
No0.5ASA0.6β	No	0.5	0.6	5	6.83
No0.5ASA0.6β	No	0.5	0.6	9	7.88
No0.5ASA0.6β	No	0.5	0.6	4	8.20
No0.5ASA0.6β	No	0.5	0.6	10	8.30
No0.5ASA0.6β	No	0.5	0.6	7	9.43
No0.5ASA0.6β	No	0.5	0.6	9	9.82
No0.5ASA0.6β	No	0.5	0.6	6	10.09
No0.5ASA0.6β	No	0.5	0.6	6	10.69
No0.5ASA0.6β	No	0.5	0.6	3	11.76
No0.5ASA0.6β	No	0.5	0.6	1	11.81
No0.5ASA0.6β	No	0.5	0.6	3	12.12
No0.5ASA0.6β	No	0.5	0.6	2	14.68
No0.5ASA0.6β	No	0.5	0.6	7	18.65
No0.5ASA0.6β	No	0.5	0.6	4	18.72
No0.5ASA0.6β	No	0.5	0.6	2	19.67
No0.5ASA0.6β	No	0.5	0.6	1	23.49
No1ASA0β	No	1.0	0.0	10	2.02
No1ASA0β	No	1.0	0.0	6	6.14

No1ASA0β	No	1.0	0.0	7	6.15
No1ASA0β	No	1.0	0.0	5	7.84
No1ASA0β	No	1.0	0.0	8	9.61
No1ASA0β	No	1.0	0.0	4	12.34
No1ASA0β	No	1.0	0.0	2	15.45
No1ASA0β	No	1.0	0.0	3	18.10
No1ASA0β	No	1.0	0.0	9	19.15
No1ASA0β	No	1.0	0.0	1	22.33
No1ASA0.6β	No	1.0	0.6	10	9.95
No1ASA0.6β	No	1.0	0.6	5	10.58
No1ASA0.6β	No	1.0	0.6	1	11.64
No1ASA0.6β	No	1.0	0.6	7	12.92
No1ASA0.6β	No	1.0	0.6	6	14.28
No1ASA0.6β	No	1.0	0.6	8	14.58
No1ASA0.6β	No	1.0	0.6	9	15.39
No1ASA0.6β	No	1.0	0.6	3	18.13
No1ASA0.6β	No	1.0	0.6	4	18.56
No1ASA0.6β	No	1.0	0.6	2	23.28
Bt0ASA0β	Bt	0.0	0.0	5	3.98
Bt0ASA0β	Bt	0.0	0.0	6	8.91
Bt0ASA0β	Bt	0.0	0.0	10	12.25
Bt0ASA0β	Bt	0.0	0.0	3	19.12
Bt0ASA0β	Bt	0.0	0.0	1	39.00
Bt0ASA0β	Bt	0.0	0.0	2	39.65
Bt0ASA0β	Bt	0.0	0.0	8	49.61
Bt0ASA0β	Bt	0.0	0.0	7	53.26
Bt0ASA0.6β	Bt	0.0	0.6	10	8.57
Bt0ASA0.6β	Bt	0.0	0.6	9	10.00
Bt0ASA0.6β	Bt	0.0	0.6	2	10.87
Bt0ASA0.6β	Bt	0.0	0.6	4	13.04
Bt0ASA0.6β	Bt	0.0	0.6	7	13.68
Bt0ASA0.6β	Bt	0.0	0.6	5	14.35
Bt0ASA0.6β	Bt	0.0	0.6	8	14.37
Bt0ASA0.6β	Bt	0.0	0.6	6	16.01
Bt0ASA0.6β	Bt	0.0	0.6	3	17.27
Bt0ASA0.6β	Bt	0.0	0.6	1	21.81
Bt0.5ASA0β	Bt	0.5	0.0	6	24.88
Bt0.5ASA0β	Bt	0.5	0.0	3	26.68
Bt0.5ASA0β	Bt	0.5	0.0	7	27.47
Bt0.5ASA0β	Bt	0.5	0.0	1	39.36
Bt0.5ASA0β	Bt	0.5	0.0	2	49.73
Bt0.5ASA0β	Bt	0.5	0.0	9	57.00
Bt0.5ASA0β	Bt	0.5	0.0	5	58.50
Bt0.5ASA0β	Bt	0.5	0.0	4	65.19
Bt0.5ASA0.6β	Bt	0.5	0.6	6	13.65
Bt0.5ASA0.6β	Bt	0.5	0.6	7	14.57
Bt0.5ASA0.6β	Bt	0.5	0.6	9	17.55
Bt0.5ASA0.6β	Bt	0.5	0.6	3	31.54
Bt0.5ASA0.6β	Bt	0.5	0.6	8	47.29
Bt0.5ASA0.6β	Bt	0.5	0.6	10	49.92

Bt0.5ASA0.6β	Bt	0.5	0.6	5	49.98
Bt0.5ASA0.6β	Bt	0.5	0.6	4	53.77
Bt1ASA0β	Bt	1.0	0.0	1	12.11
Bt1ASA0β	Bt	1.0	0.0	3	20.91
Bt1ASA0β	Bt	1.0	0.0	10	24.81
Bt1ASA0β	Bt	1.0	0.0	6	25.00
Bt1ASA0β	Bt	1.0	0.0	9	33.25
Bt1ASA0β	Bt	1.0	0.0	4	33.55
Bt1ASA0β	Bt	1.0	0.0	7	34.39
Bt1ASA0β	Bt	1.0	0.0	5	48.25
Bt1ASA0.6β	Bt	1.0	0.6	8	10.31
Bt1ASA0.6β	Bt	1.0	0.6	7	12.21
Bt1ASA0.6β	Bt	1.0	0.6	3	14.58
Bt1ASA0.6β	Bt	1.0	0.6	9	14.88
Bt1ASA0.6β	Bt	1.0	0.6	10	18.24
Bt1ASA0.6β	Bt	1.0	0.6	6	19.57
Bt1ASA0.6β	Bt	1.0	0.6	2	20.29
Bt1ASA0.6β	Bt	1.0	0.6	5	21.20
Bt1ASA0.6β	Bt	1.0	0.6	1	22.01
Bt1ASA0.6β	Bt	1.0	0.6	4	26.05

Helicoverpa sod

DIET	BT	AsA	β	REP	SOD
No1AsA0.6β	No	1	0.6	6	3.02
No1AsA0.6β	No	1	0.6	13	3.51
Bt0AsA0.6β	Bt	0	0.6	6	4.97
Bt0AsA0.6β	Bt	0	0.6	13	5.82
Bt.5ASA0β	Bt	0.5	0	4	7.14
No0AsA0.6β	No	0	0.6	6	8.27
No.5ASA0β	No	0.5	0	7	8.65
No1AsA0.6β	No	1	0.6	10	8.78
No0.5AsA0β	No	0.5	0	3	9.18
Bt0ASA0β	Bt	0	0	7	9.74
No0AsA0.6β	No	0	0.6	13	9.77
Bt.5ASA.6β	Bt	0.5	0.6	7	10.17
Bt0AsA0.6β	Bt	0	0.6	12	10.22
Bt0AsA0.6β	Bt	0	0.6	5	10.34
Bt1AsA0.6β	Bt	1	0.6	4	10.71
Bt.5ASA0β	Bt	0.5	0	7	11.06
No1AsA0.6β	No	1	0.6	3	11.29
Bt1ASA0β	Bt	1	0	7	11.56
No.5ASA.6β	No	0.5	0.6	7	11.69
No0AsA0β	No	0	0	12	11.77
No0AsA0β	No	0	0	5	12.05
Bt0AsA0.6β	Bt	0	0.6	8	12.05
Bt1AsA0.6β	Bt	1	0.6	10	12.35
No0.5AsA0β	No	0.5	0	11	12.50
Bt1AsA0.6β	Bt	1	0.6	9	12.74
No1AsA0β	No	1	0	9	12.90
No1AsA0β	No	1	0	4	13.14
No1AsA0β	No	1	0	11	13.22
No1AsA0.6β	No	1	0.6	1	13.22
Bt1AsA0.6β	Bt	1	0.6	1	13.39
Bt1AsA0.6β	Bt	1	0.6	11	13.39
No1AsA0.6β	No	1	0.6	14	13.64
Bt1AsA0.6β	Bt	1	0.6	8	13.64
No1AsA0β	No	1	0	10	13.73
No1AsA0β	No	1	0	3	13.99
No0AsA0β	No	0	0	6	14.08
No1AsA0β	No	1	0	1	14.27
No1AsA0.6β	No	1	0.6	8	14.27
No1AsA0.6β	No	1	0.6	5	14.55
Bt1AsA0.6β	Bt	1	0.6	2	14.55
Bt1AsA0.6β	Bt	1	0.6	3	14.55
No0AsA0β	No	0	0	8	14.93
Bt0AsA0.6β	Bt	0	0.6	1	15.13
Bt1AsA0.6β	Bt	1	0.6	5	15.23
Bt1AsA0.6β	Bt	1	0.6	6	15.23
No0AsA0β	No	0	0	3	15.74
No1AsA0β	No	1	0	5	15.85
No.5ASA.6β	No	0.5	0.6	9	15.86

No0AsA0β	No	0	0	1	16.06
No0.5AsA0β	No	0.5	0	5	16.17
No1AsA0β	No	1	0	8	16.28
No0.5AsA0β	No	0.5	0	4	16.40
Bt0AsA0.6β	Bt	0	0.6	11	16.51
Bt0AsA0.6β	Bt	0	0.6	14	16.62
Bt0ASA0β	Bt	0	0	2	16.94
Bt1ASA0β	Bt	1	0	9	17.07
No0AsA0β	No	0	0	10	17.09
Bt1ASA0β	Bt	1	0	6	17.12
No0AsA0β	No	0	0	2	17.21
No0AsA0β	No	0	0	13	17.21
No0.5AsA0β	No	0.5	0	1	17.21
Bt1ASA0β	Bt	1	0	2	17.22
Bt0ASA0β	Bt	0	0	1	17.29
Bt0ASA0β	Bt	0	0	6	17.39
No0.5AsA0β	No	0.5	0	10	17.45
Bt0ASA0β	Bt	0	0	5	17.45
Bt.5ASA0β	Bt	0.5	0	2	17.54
No.5ASA.6β	No	0.5	0.6	5	17.57
No.5ASA.6β	No	0.5	0.6	3	17.67
No0AsA0β	No	0	0	11	17.69
No0.5AsA0β	No	0.5	0	2	17.69
No.5ASA.6β	No	0.5	0.6	1	17.73
Bt1ASA0β	Bt	1	0	8	17.83
Bt.5ASA.6β	Bt	0.5	0.6	1	17.92
No1AsA0β	No	1	0	2	17.95
No1AsA0.6β	No	1	0.6	12	17.95
No.5ASA0β	No	0.5	0	4	17.98
No0.5AsA0β	No	0.5	0	8	18.07
Bt.5ASA0β	Bt	0.5	0	1	18.07
No.5ASA0β	No	0.5	0	2	18.17
No1AsA0β	No	1	0	12	18.20
No0.5AsA0β	No	0.5	0	12	18.20
Bt.5ASA0β	Bt	0.5	0	5	18.33
Bt0AsA0.6β	Bt	0	0.6	4	18.33
Bt1ASA0β	Bt	1	0	4	18.35
Bt1ASA0β	Bt	1	0	1	18.40
No.5ASA.6β	No	0.5	0.6	8	18.41
No0AsA0β	No	0	0	9	18.60
No0AsA0.6β	No	0	0.6	11	18.60
No1AsA0.6β	No	1	0.6	9	18.60
Bt.5ASA0β	Bt	0.5	0	6	18.65
No0AsA0β	No	0	0	4	18.73
Bt1ASA0β	Bt	1	0	5	18.73
No.5ASA0β	No	0.5	0	3	18.74
Bt.5ASA.6β	Bt	0.5	0.6	5	18.75
Bt1AsA0.6β	Bt	1	0.6	13	18.87
Bt1AsA0.6β	Bt	1	0.6	12	19.29
No0.5AsA0β	No	0.5	0	9	19.29

Bt0ASA0β	Bt	0	0	3	19.32
Bt.5ASA.6β	Bt	0.5	0.6	3	19.42
No.5ASA0β	No	0.5	0	5	19.45
Bt.5ASA.6β	Bt	0.5	0.6	6	19.77
No1AsA0.6β	No	1	0.6	2	19.87
Bt.5ASA0β	Bt	0.5	0	8	19.96
No.5ASA.6β	No	0.5	0.6	6	20.12
Bt0AsA0.6β	Bt	0	0.6	2	20.16
No.5ASA0β	No	0.5	0	1	20.30
No.5ASA0β	No	0.5	0	6	20.36
Bt.5ASA.6β	Bt	0.5	0.6	9	20.73
Bt.5ASA.6β	Bt	0.5	0.6	2	20.91
No0AsA0.6β	No	0	0.6	3	20.94
No1AsA0.6β	No	1	0.6	11	20.94
Bt0AsA0.6β	Bt	0	0.6	9	20.94
Bt1ASA0β	Bt	1	0	3	20.97
No1AsA0β	No	1	0	14	21.10
No.5ASA.6β	No	0.5	0.6	2	21.19
Bt0ASA0β	Bt	0	0	9	21.31
Bt0ASA0β	Bt	0	0	4	21.35
Bt.5ASA0β	Bt	0.5	0	9	21.44
No1AsA0β	No	1	0	6	21.59
Bt.5ASA0β	Bt	0.5	0	3	21.65
No.5ASA.6β	No	0.5	0.6	4	21.84
Bt0ASA0β	Bt	0	0	8	22.20
Bt.5ASA.6β	Bt	0.5	0.6	4	23.49
Bt1AsA0.6β	Bt	1	0.6	14	23.75
No0AsA0.6β	No	0	0.6	1	24.34
Bt.5ASA.6β	Bt	0.5	0.6	8	24.49
No0AsA0.6β	No	0	0.6	4	24.74
No0.5AsA0β	No	0.5	0	7	24.74
No0AsA0.6β	No	0	0.6	8	25.16
No1AsA0.6β	No	1	0.6	4	25.16
No0AsA0β	No	0	0	7	26.25
No0AsA0.6β	No	0	0.6	9	26.25
No0AsA0.6β	No	0	0.6	10	26.47
No0AsA0.6β	No	0	0.6	7	26.93
No0.5AsA0β	No	0.5	0	6	26.93
No0AsA0.6β	No	0	0.6	14	27.17
No1AsA0β	No	1	0	13	27.90
No0AsA0.6β	No	0	0.6	5	28.14
Bt0AsA0.6β	Bt	0	0.6	3	28.91
No.5ASA0β	No	0.5	0	8	29.68
Bt0AsA0.6β	Bt	0	0.6	10	30.54
No.5ASA0β	No	0.5	0	9	30.65
No0.5AsA0β	No	0.5	0	14	31.12
No0AsA0β	No	0	0	14	32.64
No0AsA0.6β	No	0	0.6	12	32.64
No1AsA0β	No	1	0	7	34.97
No1AsA0.6β	No	1	0.6	7	36.05

No0.5AsA0β	No	0.5	0	13	36.05
Bt0AsA0.6β	Bt	0	0.6	7	38.38
No0AsA0.6β	No	0	0.6	2	40.51
Bt1AsA0.6β	Bt	1	0.6	7	55.50

helicoverpa cat

**Diet**	**BT**	**ASA**	**β**	**rep**	**HA CAT**
Bt0ASA0β	Bt	0	0	1	52.20
Bt.5ASA0β	Bt	0.5	0	1	47.93
Bt.5ASA0.6β	Bt	0.5	0.6	1	47.34
Bt1ASA0β	Bt	1	0	1	47.23
No0.5ASA0.6	No	0.5	0.6	2	42.97
Bt.5ASA0.6β	Bt	0.5	0.6	2	40.79
No0.5ASA0β	No	0.5	0	4	32.51
No1ASA0.6β	No	1	0.6	1	31.98
No0.5ASA0.6	No	0.5	0.6	4	31.35
No0.5ASA0β	No	0.5	0	1	31.30
No0.5ASA0β	No	0.5	0	5	29.93
No1ASA0β	No	1	0	3	29.06
No0.5ASA0β	No	0.5	0	6	28.64
No1ASA0β	No	1	0	3	28.62
No0.5ASA0β	No	0.5	0	2	28.15
No1ASA0β	No	1	0	5	27.18
No0.5ASA0.6	No	0.5	0.6	6	26.97
No0.5ASA0.6	No	0.5	0.6	7	26.97
No0.5ASA0.6	No	0.5	0.6	1	26.83
No0ASA0β	No	0	0	2	26.69
No1ASA0β	No	1	0	4	26.53
No0.5ASA0β	No	0.5	0	5	26.45
No0ASA0β	No	0	0	2	26.35
No0.5ASA0.6	No	0.5	0.6	5	26.25
No0.5ASA0β	No	0.5	0	5	25.68
No1ASA0β	No	1	0	5	25.59
No0ASA0β	No	0	0	4	25.05
No0.5ASA0β	No	0.5	0	3	25.05
No1ASA0β	No	1	0	4	24.91
No0.5ASA0β	No	0.5	0	1	24.90
No0ASA0β	No	0	0	1	24.89
No0.5ASA0β	No	0.5	0	1	24.44
No0ASA0β	No	0	0	1	24.06
No1ASA0β	No	1	0	1	23.87
No0ASA0.6β	No	0	0.6	1	23.80
No0ASA0.6β	No	0	0.6	1	23.53
No1ASA0.6β	No	1	0.6	1	23.38
No0.5ASA0β	No	0.5	0	4	23.17
No1ASA0.6β	No	1	0.6	2	22.98
No0.5ASA0.6	No	0.5	0.6	3	22.95
No1ASA0β	No	1	0	1	22.82
No0ASA0.6β	No	0	0.6	2	22.73
No1ASA0.6β	No	1	0.6	2	22.70
No0.5ASA0.6	No	0.5	0.6	9	22.31
No0ASA0β	No	0	0	4	22.19
No1ASA0β	No	1	0	6	22.15
No1ASA0.6β	No	1	0.6	5	22.11
No0.5ASA0β	No	0.5	0	2	21.77

No0.5ASA0β	No	0.5	0	4	21.65
No0.5ASA0β	No	0.5	0	3	21.55
No1ASA0β	No	1	0	6	21.52
No0.5ASA0β	No	0.5	0	3	21.49
No0.5ASA0.6	No	0.5	0.6	10	21.24
No0ASA0.6β	No	0	0.6	2	20.71
No0.5ASA0β	No	0.5	0	2	20.68
No1ASA0β	No	1	0	2	20.60
No0.5ASA0β	No	0.5	0	11	20.24
No1ASA0.6β	No	1	0.6	4	19.77
No0ASA0β	No	0	0	3	19.52
No1ASA0.6β	No	1	0.6	5	19.52
No0ASA0β	No	0	0	6	19.39
No1ASA0β	No	1	0	2	19.25
No0ASA0β	No	0	0	6	19.06
No0ASA0β	No	0	0	3	18.80
No0ASA0.6β	No	0	0.6	3	18.74
No0.5ASA0.6	No	0.5	0.6	11	18.46
No1ASA0.6β	No	1	0.6	4	18.29
No0ASA0β	No	0	0	5	18.07
No0ASA0β	No	0	0	5	17.78
No0ASA0.6β	No	0	0.6	3	17.28
No0ASA0.6β	No	0	0.6	4	17.24
Bt1ASA0.6β	Bt	1	0.6	3	16.00
No0ASA0.6β	No	0	0.6	4	15.89
Bt1ASA0.6β	Bt	1	0.6	3	14.72
No1ASA0.6β	No	1	0.6	3	14.33
No0ASA0.6β	No	0	0.6	5	13.99
No0ASA0.6β	No	0	0.6	5	13.60
No1ASA0.6β	No	1	0.6	3	13.52
Bt1ASA0.6β	Bt	1	0.6	5	11.87
No0.5ASA0β	No	0.5	0	8	11.74
Bt1ASA0.6β	Bt	1	0.6	5	11.27
No0.5ASA0.6	No	0.5	0.6	12	10.58
No0.5ASA0β	No	0.5	0	10	10.31
Bt1ASA0.6β	Bt	1	0.6	4	9.54
Bt1ASA0β	Bt	1	0	5	9.42
Bt1ASA0.6β	Bt	1	0.6	1	9.35
No0.5ASA0β	No	0.5	0	12	9.32
Bt1ASA0.6β	Bt	1	0.6	4	9.14
No0.5ASA0β	No	0.5	0	7	8.87
Bt0ASA0.6β	Bt	0	0.6	4	8.71
Bt0ASA0.6β	Bt	0	0.6	3	8.42
Bt0ASA0.6β	Bt	0	0.6	3	8.40
Bt0ASA0.6β	Bt	0	0.6	4	8.30
Bt0ASA0β	Bt	0	0	7	8.10
Bt0ASA0.6β	Bt	0	0.6	2	7.73
No0.5ASA0.6	No	0.5	0.6	8	7.57
Bt1ASA0.6β	Bt	1	0.6	1	7.53
Bt1ASA0β	Bt	1	0	4	7.48

Bt0ASA0.6β	Bt	0	0.6	1	7.44
Bt0ASA0.6β	Bt	0	0.6	5	7.32
Bt0ASA0.6β	Bt	0	0.6	1	7.29
Bt.5ASA0β	Bt	0.5	0	2	7.22
Bt.5ASA0.6β	Bt	0.5	0.6	11	7.19
Bt0ASA0.6β	Bt	0	0.6	2	7.08
Bt0ASA0.6β	Bt	0	0.6	5	6.69
Bt1ASA0β	Bt	1	0	2	6.60
Bt0ASA0β	Bt	0	0	4	5.80
Bt0ASA0β	Bt	0	0	3	5.67
No0.5ASA0β	No	0.5	0	9	5.67
Bt.5ASA0.6β	Bt	0.5	0.6	4	5.48
Bt.5ASA0β	Bt	0.5	0	4	5.19
Bt0ASA0β	Bt	0	0	2	4.91
Bt1ASA0β	Bt	1	0	12	4.48
Bt0ASA0β	Bt	0	0	10	4.36
Bt.5ASA0β	Bt	0.5	0	5	4.27
Bt1ASA0.6β	Bt	1	0.6	2	4.23
Bt.5ASA0.6β	Bt	0.5	0.6	7	4.18
Bt.5ASA0.6β	Bt	0.5	0.6	5	4.14
Bt.5ASA0β	Bt	0.5	0	3	4.09
Bt.5ASA0.6β	Bt	0.5	0.6	3	3.97
Bt1ASA0β	Bt	1	0	10	3.94
Bt1ASA0.6β	Bt	1	0.6	2	3.76
Bt1ASA0β	Bt	1	0	6	3.60
Bt.5ASA0β	Bt	0.5	0	6	3.10
Bt.5ASA0β	Bt	0.5	0	8	2.37
Bt0ASA0β	Bt	0	0	6	2.09
Bt.5ASA0.6β	Bt	0.5	0.6	6	2.03
Bt.5ASA0β	Bt	0.5	0	9	2.01
Bt1ASA0β	Bt	1	0	7	2.00
Bt.5ASA0β	Bt	0.5	0	7	1.92
Bt0ASA0β	Bt	0	0	8	1.84
Bt.5ASA0.6β	Bt	0.5	0.6	10	1.82
Bt1ASA0β	Bt	1	0	3	1.79
Bt0ASA0β	Bt	0	0	5	1.49
Bt.5ASA0.6β	Bt	0.5	0.6	8	1.20
Bt1ASA0β	Bt	1	0	8	1.14
Bt.5ASA0.6β	Bt	0.5	0.6	9	0.70
Bt1ASA0β	Bt	1	0	9	0.62
Bt0ASA0β	Bt	0	0	9	0.57
Bt.5ASA0β	Bt	0.5	0	10	0.54

Helicoverpa gst

Diet	BT	ASA	β	Rep	Ha GST
No0ASA0β	No	0	0	1	15.17
No0ASA0β	No	0	0	2	10.95
No0ASA0β	No	0	0	3	15.03
No0ASA0β	No	0	0	4	20.81
No0ASA0β	No	0	0	5	10.51
No0ASA0β	No	0	0	6	11.53
No0ASA0β	No	0	0	7	5.37
No0ASA0β	No	0	0	8	2.76
No0ASA0β	No	0	0	9	0.99
No0ASA0.6β	No	0	0.6	1	6.29
No0ASA0.6β	No	0	0.6	2	4.29
No0ASA0.6β	No	0	0.6	3	5.00
No0ASA0.6β	No	0	0.6	4	5.20
No0ASA0.6β	No	0	0.6	5	3.60
No0ASA0.6β	No	0	0.6	6	4.28
No0ASA0.6β	No	0	0.6	7	3.50
No0ASA0.6β	No	0	0.6	8	4.83
No0ASA0.6β	No	0	0.6	9	5.11
No0ASA0.6β	No	0	0.6	10	1.22
No0.5ASA0β	No	0.5	0	1	3.54
No0.5ASA0β	No	0.5	0	2	2.89
No0.5ASA0β	No	0.5	0	3	6.36
No0.5ASA0β	No	0.5	0	4	4.91
No0.5ASA0β	No	0.5	0	5	10.49
No0.5ASA0β	No	0.5	0	6	4.41
No0.5ASA0β	No	0.5	0	7	15.13
No0.5ASA0β	No	0.5	0	8	8.80
No0.5ASA0β	No	0.5	0	9	5.03
No0.5ASA0β	No	0.5	0	10	0.93
No0.5ASA0β	No	0.5	0	11	2.98
No0.5ASA0β	No	0.5	0	12	2.07
No0.5ASA0β	No	0.5	0	13	1.02
No0.5ASA0β	No	0.5	0	14	4.68
No0.5ASA0β	No	0.5	0	15	6.15
No0.5ASA0β	No	0.5	0	16	4.45
No0.5ASA0β	No	0.5	0	17	7.06
No0.5ASA0β	No	0.5	0	18	7.04
No0.5ASA0β	No	0.5	0	19	4.59
No0.5ASA0β	No	0.5	0	20	4.31
No0.5ASA0β	No	0.5	0	21	3.86
No0.5ASA0β	No	0.5	0	22	4.97
No0.5ASA0β	No	0.5	0	23	2.00
No0.5ASA0β	No	0.5	0	24	2.24
No0.5ASA0β	No	0.5	0	25	2.07
No0.5ASA0.6β	No	0.5	0.6	1	9.52
No0.5ASA0.6β	No	0.5	0.6	2	1.63
No0.5ASA0.6β	No	0.5	0.6	3	0.97
No0.5ASA0.6β	No	0.5	0.6	4	3.39

No0.5ASA0.6β	No	0.5	0.6	5	6.57
No0.5ASA0.6β	No	0.5	0.6	6	2.75
No0.5ASA0.6β	No	0.5	0.6	7	2.87
No0.5ASA0.6β	No	0.5	0.6	8	2.41
No0.5ASA0.6β	No	0.5	0.6	9	5.03
No0.5ASA0.6β	No	0.5	0.6	10	2.77
No0.5ASA0.6β	No	0.5	0.6	11	6.61
No0.5ASA0.6β	No	0.5	0.6	12	4.34
No0.5ASA0.6β	No	0.5	0.6	13	6.86
No0.5ASA0.6β	No	0.5	0.6	14	2.50
No0.5ASA0.6β	No	0.5	0.6	15	1.63
No1.0ASA0β	No	1	0	1	9.00
No1.0ASA0β	No	1	0	2	6.33
No1.0ASA0β	No	1	0	3	5.63
No1.0ASA0β	No	1	0	4	5.84
No1.0ASA0β	No	1	0	5	6.90
No1.0ASA0β	No	1	0	6	6.74
No1.0ASA0β	No	1	0	7	8.17
No1.0ASA0β	No	1	0	8	3.11
No1.0ASA0β	No	1	0	9	2.99
No1.0ASA0β	No	1	0	10	1.44
No1.0ASA0.6β	No	1	0.6	1	3.10
No1.0ASA0.6β	No	1	0.6	2	7.96
No1.0ASA0.6β	No	1	0.6	3	5.74
No1.0ASA0.6β	No	1	0.6	4	6.34
No1.0ASA0.6β	No	1	0.6	5	10.18
No1.0ASA0.6β	No	1	0.6	6	3.98
No1.0ASA0.6β	No	1	0.6	7	8.91
No1.0ASA0.6β	No	1	0.6	8	2.14
No1.0ASA0.6β	No	1	0.6	9	3.96
No1.0ASA0.6β	No	1	0.6	10	3.00
Bt0ASA0β	Bt	0	0	1	8.00
Bt0ASA0β	Bt	0	0	2	12.32
Bt0ASA0β	Bt	0	0	3	7.56
Bt0ASA0β	Bt	0	0	4	11.31
Bt0ASA0β	Bt	0	0	5	9.72
Bt0ASA0β	Bt	0	0	6	4.40
Bt0ASA0β	Bt	0	0	7	8.00
Bt0ASA0β	Bt	0	0	8	24.17
Bt0ASA0β	Bt	0	0	9	2.11
Bt0ASA0β	Bt	0	0	10	5.15
Bt0ASA0β	Bt	0	0	11	3.30
Bt0ASA0β	Bt	0	0	12	5.47
Bt0ASA0β	Bt	0	0	13	1.92
Bt0ASA0β	Bt	0	0	14	12.56
Bt0ASA0β	Bt	0	0	15	12.32
Bt0ASA0.6β	Bt	0	0.6	1	5.42
Bt0ASA0.6β	Bt	0	0.6	2	13.07
Bt0ASA0.6β	Bt	0	0.6	3	5.26
Bt0ASA0.6β	Bt	0	0.6	4	7.86

Bt0ASA0.6β	Bt	0	0.6	5	11.19
Bt0ASA0.6β	Bt	0	0.6	6	5.16
Bt0ASA0.6β	Bt	0	0.6	7	11.47
Bt0ASA0.6β	Bt	0	0.6	8	6.21
Bt0ASA0.6β	Bt	0	0.6	9	7.26
Bt0ASA0.6β	Bt	0	0.6	10	1.24
Bt0.5ASA0β	Bt	0.5	0	1	2.25
Bt0.5ASA0β	Bt	0.5	0	2	6.41
Bt0.5ASA0β	Bt	0.5	0	3	3.70
Bt0.5ASA0β	Bt	0.5	0	4	6.03
Bt0.5ASA0β	Bt	0.5	0	5	11.41
Bt0.5ASA0β	Bt	0.5	0	6	9.25
Bt0.5ASA0β	Bt	0.5	0	7	10.46
Bt0.5ASA0β	Bt	0.5	0	8	20.67
Bt0.5ASA0β	Bt	0.5	0	9	4.10
Bt0.5ASA0β	Bt	0.5	0	10	5.30
Bt0.5ASA0β	Bt	0.5	0	11	11.88
Bt0.5ASA0β	Bt	0.5	0	12	10.17
Bt0.5ASA0β	Bt	0.5	0	13	9.63
Bt0.5ASA0β	Bt	0.5	0	14	4.46
Bt0.5ASA0β	Bt	0.5	0	15	6.41
Bt0.5ASA0.6β	Bt	0.5	0.6	1	8.71
Bt0.5ASA0.6β	Bt	0.5	0.6	2	4.35
Bt0.5ASA0.6β	Bt	0.5	0.6	3	3.59
Bt0.5ASA0.6β	Bt	0.5	0.6	4	15.49
Bt0.5ASA0.6β	Bt	0.5	0.6	5	2.44
Bt0.5ASA0.6β	Bt	0.5	0.6	6	2.74
Bt0.5ASA0.6β	Bt	0.5	0.6	7	6.06
Bt0.5ASA0.6β	Bt	0.5	0.6	8	3.32
Bt0.5ASA0.6β	Bt	0.5	0.6	9	2.72
Bt0.5ASA0.6β	Bt	0.5	0.6	10	1.43
Bt0.5ASA0.6β	Bt	0.5	0.6	11	2.05
Bt0.5ASA0.6β	Bt	0.5	0.6	12	2.51
Bt0.5ASA0.6β	Bt	0.5	0.6	13	1.63
Bt0.5ASA0.6β	Bt	0.5	0.6	14	4.34
Bt0.5ASA0.6β	Bt	0.5	0.6	15	4.35
Bt1ASA0β	Bt	1	0	1	3.79
Bt1ASA0β	Bt	1	0	2	6.61
Bt1ASA0β	Bt	1	0	3	3.08
Bt1ASA0β	Bt	1	0	4	13.06
Bt1ASA0β	Bt	1	0	5	4.54
Bt1ASA0β	Bt	1	0	6	16.34
Bt1ASA0β	Bt	1	0	7	10.43
Bt1ASA0β	Bt	1	0	8	35.03
Bt1ASA0β	Bt	1	0	9	2.49
Bt1ASA0β	Bt	1	0	10	3.97
Bt1ASA0β	Bt	1	0	11	7.02
Bt1ASA0β	Bt	1	0	12	5.94
Bt1ASA0β	Bt	1	0	13	10.45
Bt1ASA0β	Bt	1	0	14	6.59

Bt1ASA0β	Bt	1	0	15	6.61
Bt1.0ASA0.6β	Bt	1	0.6	1	8.33
Bt1.0ASA0.6β	Bt	1	0.6	2	11.92
Bt1.0ASA0.6β	Bt	1	0.6	3	4.40
Bt1.0ASA0.6β	Bt	1	0.6	4	8.37
Bt1.0ASA0.6β	Bt	1	0.6	5	19.50
Bt1.0ASA0.6β	Bt	1	0.6	6	7.05
Bt1.0ASA0.6β	Bt	1	0.6	7	8.50
Bt1.0ASA0.6β	Bt	1	0.6	8	4.40
Bt1.0ASA0.6β	Bt	1	0.6	9	3.25
Bt1.0ASA0.6β	Bt	1	0.6	10	1.17
